# Environment selected microbial function rather than taxonomic species in a plateau saline-alkaline wetland

**DOI:** 10.1128/aem.02206-24

**Published:** 2025-07-03

**Authors:** Hongjie Zhang, Dayong Zhao, Qinglong L. Wu, Jin Zeng

**Affiliations:** 1 State Key Laboratory of Water Disaster Prevention, Hohai University 12462, Nanjing, Jiangsu, China; 2 State Key Laboratory of Lake and Watershed Science for Water Security, Nanjing Institute of Geography and Limnology, Chinese Academy of Sciences 66289, Nanjing, Jiangsu, China; 3 Center for Evolution and Conservation Biology, Southern Marine Science and Engineering Guangdong Laboratory (Guangzhou) 606379 , Guangzhou, Guangdong, China; 4 Sino-Danish Centre for Education and Research, University of Chinese Academy of Sciences 74519, Beijing, China; 5 Poyang Lake Wetland Research Station, Nanjing Institute of Geography and Limnology, Chinese Academy of Sciences 66289, Jiujiang, Jiangxi, China; Colorado School of Mines, Golden, Colorado, USA

**Keywords:** community structure, functional compositions, metagenomic sequencing, methane/nitrogen/sulfur cycling, plateau saline-alkaline wetlands

## Abstract

Comprehending the microbial community in plateau saline-alkaline wetlands, an understudied and vulnerable ecosystem, is vital for predicting ecosystem functions within the context of global climate change. Despite the rapid shrinkage and potential drying up of some of these wetlands, our knowledge of the microbial community in this ecosystem remains fragmented. Here, we utilized metagenomic sequencing to investigate the distribution of methane, nitrogen, and sulfur cycling genes/pathways and formation mechanism of microbial communities across sediment, surface rhizosphere soils (R_surface_), subsurface rhizosphere soils (R_subsurface_), surface bulk soils (B_surface_), and subsurface bulk soils (B_subsurface_) in Cuochuolong Wetland, a typical saline-alkaline wetland located in the Tibetan Plateau. The results showed that sediment exhibited relatively higher functional potentials for methanogenesis but lower potentials for methane oxidation. Denitrification and dissimilatory sulfate reduction potentials increased with decreasing salinity across the five habitats, following the trend: sediment <R_surface_ < R_subsurface_ <B_surface_ < B_subsurface_. The taxonomic compositions of microbial communities varied more dramatically, yet functional genes distributed relatively evenly, indicating functional redundancy. Greater determinacy was observed in functional compositions, whereas taxonomic compositions exhibited more stochasticity. Similar patterns were observed within individual habitats, with the relative importance of deterministic processes increasing as salinity levels increased across the five habitats. Additionally, 188 non-redundant medium- and high-quality metagenome-assembled genomes (MAGs) were reconstructed, with 18 MAGs containing the *nod* gene, a marker gene of disproportionation of nitric oxide. This study provided a novel perspective on the formation mechanism of microbial community by emphasizing the deterministic selection of extreme environments on microbial function.

**IMPORTANCE:**

Understanding the formation mechanism of microbial communities is a central goal in ecology. However, our understanding of microbial community remains fragmented in plateau saline-alkaline wetlands, despite their unique status as a vulnerable ecosystem characterized by high altitude, low disturbance, high salinity, sensitivity to global climate change, and localized shrinkage in some areas. Furthermore, previous studies on community formation mechanism have predominantly focused on microbial taxonomic structure, neglecting their functional compositions. Beyond providing a comprehensive understanding of the distribution patterns of methane, nitrogen, and sulfur cycling microbial communities within plateau saline-alkaline wetland, this study offers a novel perspective on formation mechanism of microbial community by emphasizing the deterministic selection of extreme environment on microbial function. This study also expands our comprehension of the diversity of microbes containing the *nod* gene, which may substantially contribute to global methane and nitrogen budgets.

## INTRODUCTION

The plateau saline-alkaline wetland represents a unique ecosystem characterized by high altitude, intense ultraviolet radiation, high salinity, and sensitivity to global climate change (1). As an extreme ecosystem, it forms a wide variety of harsh conditions and provides unique and diverse ecological niches for life on the plateau (2). Microbial communities are the vital life forms in harsh ecosystems and the sensitive indicator for environmental change, which drive biogeochemical cycling, perform essential ecosystem functions, and maintain ecosystem stability (3). Nevertheless, the microbial communities inhabiting the plateau saline-alkaline wetland are considerably less understood compared with those present in plain ecosystems. The study of the formation mechanism (encompassing both deterministic and stochastic processes), composition, and function is a core element of microbial ecology (4–6). Comprehending the formation mechanism of microbial communities in plateau saline-alkaline wetland, an understudied and unique ecosystem, provides a unique perspective to understand the composition and distribution of microbes and is vital for predicting harsh ecosystem functions within the context of global climate change (7). Nonetheless, some of plateau saline-alkaline wetlands, which are primarily supplied by precipitation, have been shrinking due to climate change (8). For instance, Cuochuolong wetland, a representative saline-alkaline wetland on the Tibetan Plateau, has been rapidly shrinking since 2006 and could dry up within approximately 50 years if the shrinkage rate and the speed of climate change observed over the past 40 years continue (9). This rapid shrinkage of plateau wetlands is driving vital environmental degradation, including accelerated salinization and biodiversity loss (10, 11). Notably, methane-cycling, nitrogen-transforming, and sulfur-cycling microbes dominate the functional guilds mediating carbon and nutrient fluxes in these shrinking wetlands (12–14). Therefore, understanding the formation mechanism of these keystone microbial communities is crucial and urgent, as their shifts could alter the biogeochemical functions of shrinking wetlands.

Although it is generally accepted that deterministic and stochastic processes synergistically drove microbial community compositions, their relative importance remains inadequately constrained, especially in inaccessible habitats, such as plateau saline-alkaline wetlands (15–17). As an extreme ecosystem with limited accessibility and low disturbance, plateau saline-alkaline wetland provides an ideal area for studying the formation mechanism of microbial communities involved in biogeochemical cycling (2). Moreover, previous studies elucidating the community formation mechanism predominantly concentrated on microbial taxonomic structure, ignoring their functional compositions (18, 19). The advances in metagenomic sequencing enable us to comprehensively relate the microbial functions involved in the biogeochemical cycling with formation mechanism of microbial community (20–22). Emerging metagenomic evidence has indicated that environmental factors typically exert stronger selective pressures on functional composition of microbial communities than on their taxonomic structure (23, 24). Furthermore, many phylogenetically distinct taxa are capable of encoding similar metabolic functions, highlighting a widespread phenomenon of functional redundancy (25, 26). Based on these insights, we hypothesized that the extreme environment of plateau saline-alkaline wetland deterministically selected microbial functions rather than the taxonomic groups, with functional redundancy underlying the stochastic taxonomic community compositions.

In the plateau saline-alkaline wetland, sediment (chronically submerged by saltwater), rhizosphere soils (non-submerged soil adjacent to the roots of living plants), and bulk soils (non-submerged soil unaffected by plants) represent three distinct habitats displaying a salinity gradient (27). Sediments generally exhibit the highest salinity due to direct exposure to saltwater, rhizosphere soils have slightly lower salinity that supports salt-tolerant plants, while bulk soils typically show the lowest salinity. Additionally, the surface and subsurface layers constituted distinct habitats with differing physicochemical properties, including salinity, oxygen availability, and plant-microbe interactions (28). However, knowledge about the microbial community formation mechanism and functional genes distribution related to methane, nitrogen, and sulfur cycling—key elements for life—remains fragmented among these contrasting habitats within the plateau saline-alkaline wetland. Previous studies have shown that community compositions and microbial function genes related to biogeochemical cycling would respond to environmental changes, such as salinity levels, water saturation, and plant-microbe interactions (29–32). Therefore, we hypothesize that microbial community formation mechanism and functional gene distribution associated with methane, nitrogen, and sulfur cycling would vary among these distinct habitats within the plateau saline-alkaline wetland. Characterizing the formation mechanism of microbial communities and associated biogeochemical processes across these diverse habitats is crucial for expanding our knowledge about the microbial ecology in this underexplored ecosystem (33).

The dismutation of nitric oxide into dinitrogen and oxygen is a novel biogeochemical pathway, which establishes a vital link between the carbon and nitrogen cycles (34). This microbially mediated process potentially mitigates the greenhouse effect by simultaneously reducing methane emissions and preventing the production of nitrous oxide (35). This reaction was proposed to be catalyzed by nitric oxide dismutase, encoded by the *nod* gene, which was initially thought to be exclusive to *Candidatus Methylomirabilis oxyfera—*a nitrite-dependent anaerobic methanotroph within the NC10 phylum (36). Recent advances in metagenome-assembled genome (MAG) analyses have expanded our knowledge about the distribution of the *nod* gene, revealing its presence across phylogenetically diverse microbial lineages, including Alphaproteobacteria, Gammaproteobacteria, and Planctomycetia (37). Moreover, *nod* sequences have been identified in diverse environments, including marine, agricultural soils, and lake sediments (38, 39). Despite these discoveries, direct evidence confirming the presence of microbes containing the *nod* gene in plateau saline-alkaline wetlands remains limited. Given the dynamic oxic-anoxic interfaces and limited bioavailable substrates in these wetlands, they are theoretically conducive to nitric oxide dismutation. With the advancements in binning analysis and omics databases, it is anticipated that systematic exploration of this understudied ecosystem will uncover previously unrecognized microbial diversity harboring the *nod* gene. An enhanced comprehension of the *nod* gene carries significant implications, as it may substantially contribute to global methane and nitrogen budgets, especially in the plateau saline-alkaline wetlands, which are sensitive to global climate change (40).

The Cuochuolong Wetland, a typical saline-alkaline wetland located on the Tibetan Plateau and with an average altitude exceeding 4,600 m, is highly vulnerable and could potentially dry up within the next 50 years if the speed of climate change observed over the past 40 years continues (9, 41). This potential drying of Cuochuolong Wetland is expected to result in severe environmental consequences, particularly accelerated salinization and biodiversity loss on the Tibetan Plateau. Therefore, understanding the formation mechanism of keystone microbial communities in these shrinking wetlands is vital and urgent. In the present study, we aim to understand the distribution of methane, nitrogen, and sulfur cycling genes and pathways, as well as the potential formation mechanism of these microbial communities in plateau saline-alkaline wetlands. Specifically, we aim to address the following scientific questions: (i) What are the distribution patterns of the taxonomic group and functional genes/pathways of microbial community involved in methane, nitrogen, and sulfur cycling across distinct habitats within the plateau saline-alkaline wetland? (ii) How do deterministic and stochastic processes contribute to shaping taxonomic and functional compositions of microbial communities within the plateau saline-alkaline wetland? (iii) Are there any microbes containing the *nod* gene that play a potential role in mitigating the greenhouse effect by simultaneously reducing methane emissions and preventing the production of nitrous oxide?

## RESULTS

### Physicochemical characteristics of the five habitats in Cuochuolong wetland

To test our hypothesis, we collected sediment, surface rhizosphere soils (R_surface_), subsurface rhizosphere soils (R_subsurface_), surface bulk soils (B_surface_), and subsurface bulk soils (B_subsurface_) samples from the Cuochuolong Wetland, which exhibited a salinity gradient.

**Fig 1:**
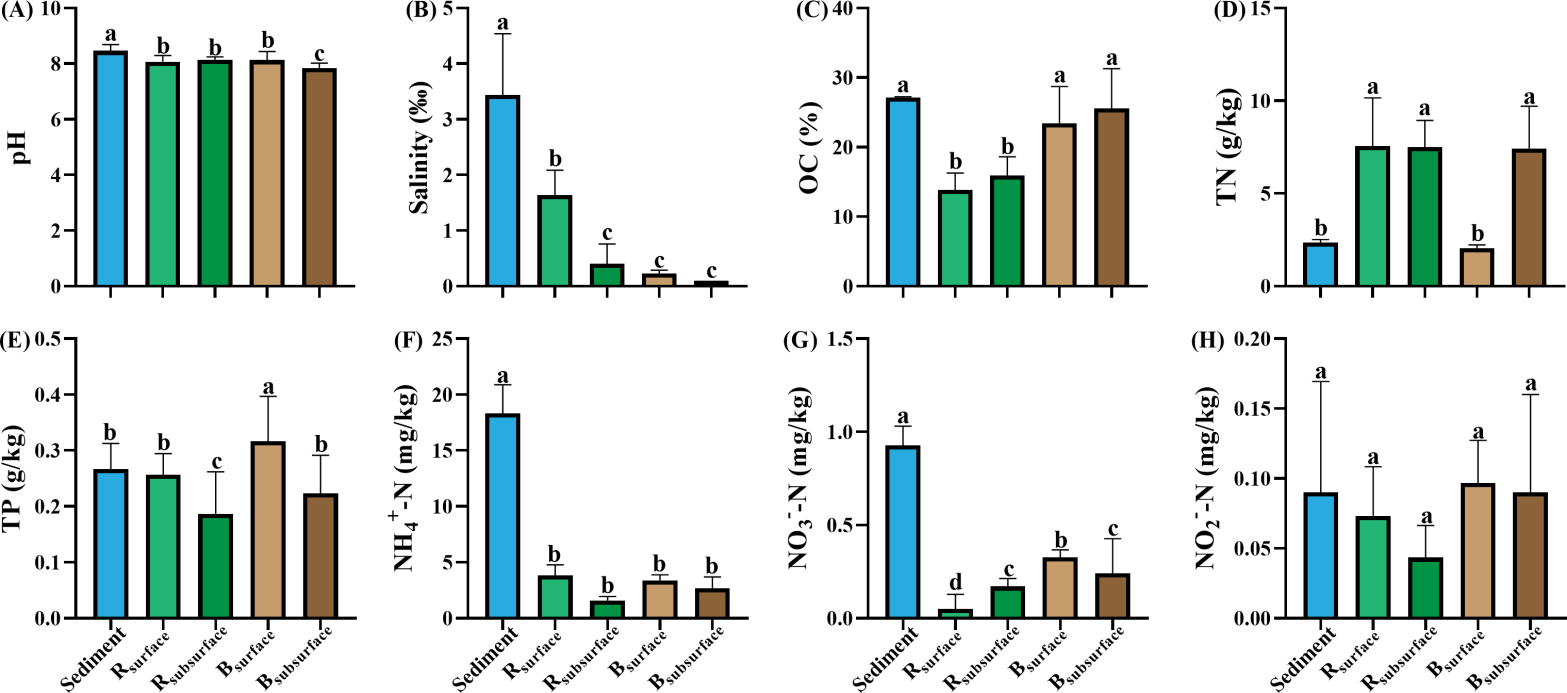
Physicochemical properties of sediment, rhizosphere soils, and bulk soils. (A) pH, (B) salinity, (C) organic carbon (OC), (D) total nitrogen (TN), (E) total phosphorus (TP), (F) ammonia nitrogen (NH_4_^+^-N), (G) nitrate nitrogen (NO_3_^-^-N), (H) nitrite nitrogen (NO_2_^-^-N). Different letters above the boxes indicated significant difference (*P* < 0.05) among habitats according to the one-way ANOVA. Sediment: sediment samples; R_surface_, surface samples of the rhizosphere soils; R_subsurface_, subsurface samples of the rhizosphere soils; B_surface_, surface samples of the bulk soils; B_subsurface_, subsurface samples of the bulk soils.

Salinity concentrations ranged from 0.1‰ to 4.6‰, exhibiting a decreasing gradient across habitats: sediment >R_surface_ > R_subsurface_ >B_surface_ > B_subsurface_ (Fig. 1). The pH, salinity, OC, NH_4_^+^-N, and NO_3_^-^-N levels of sediment were significantly higher than those of other habitats, while the concentration of TN in the sediment was significantly lower compared with that in other habitats (*P* < 0.05). For rhizosphere and bulk soils, the concentration of TP was significantly (*P* < 0.05) higher in the surface layer compared with the subsurface layer.

### Taxonomic structures of microbial communities across the five habitats

In all five habitats, bacteria were the most dominant microbes (Fig. S1). A total of 73 microbial phyla/subphyla were identified across the five habitats (Table S4), with all dominant phyla/subphyla (relative abundance >1%) presented in Fig. S2. Actinobacteria was the most abundant phylum in all five habitats, accounting for mean relative abundances of 37.35% in sediment, 47.87% in R_surface_, 34.90% in R_subsurface_, 34.87% in B_surface_, and 24.63% in B_subsurface_, respectively. Other dominant phyla included Alphaproteobacteria, Betaproteobacteria, Gammaproteobacteria, Deltaproteobacteria, Bacteroidetes, Firmicutes, and Streptophyta.

### Distribution of key functional genes and pathways of microbially driven methane, nitrogen, and sulfur cycling

To comprehensively understand the distribution patterns of functional genes and pathways of microbially driven methane, nitrogen, and sulfur cycling, we analyzed functional profiles based on the metagenomic sequencing data, and a notable divergence of these metabolic patterns among the five habitats was observed.

#### Methane cycling

**Fig 2:**
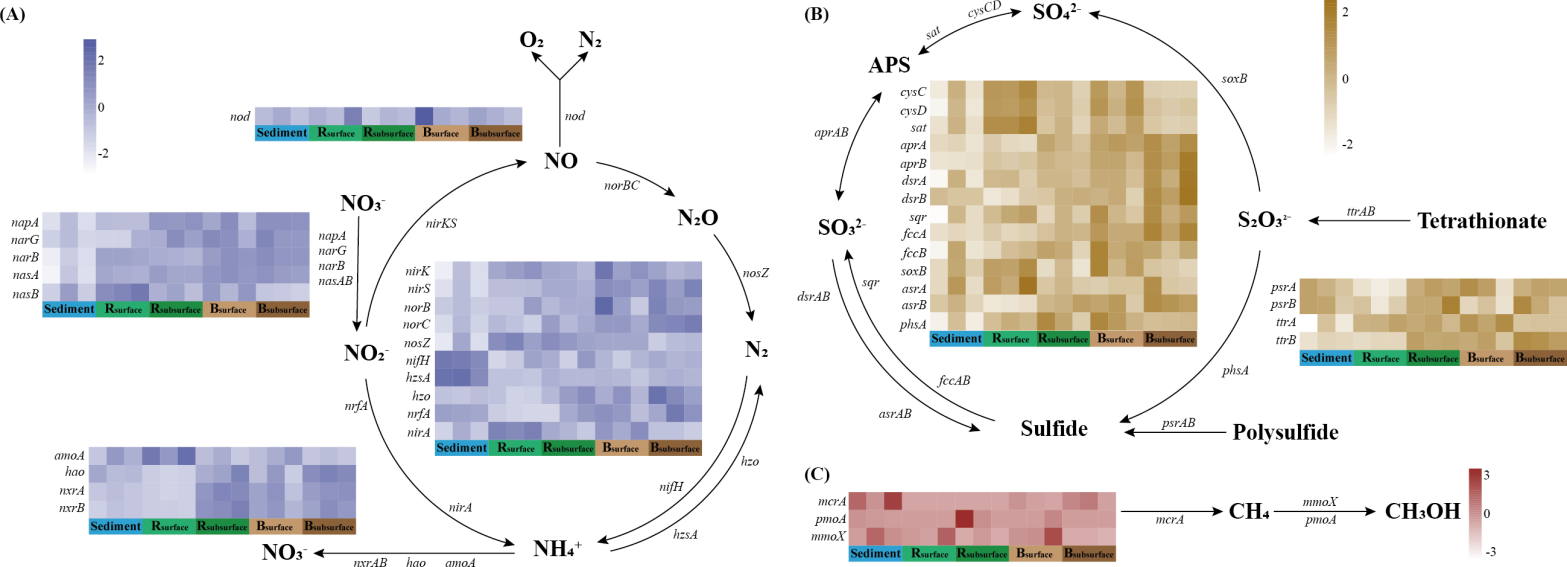
Heatmaps of normalized relative abundances of key genes involved in nitrogen (A), sulfur (B), and methane (C) cycles. The normalization process involved subtracting the mean of each row from each value and then dividing it by the standard deviation of that row. Sediment: sediment samples; R_surface_, surface samples of the rhizosphere soils; R_subsurface_, subsurface samples of the rhizosphere soils; B_surface_, surface samples of the bulk soils; B_subsurface_, subsurface samples of the bulk soils.

**Fig 3:**
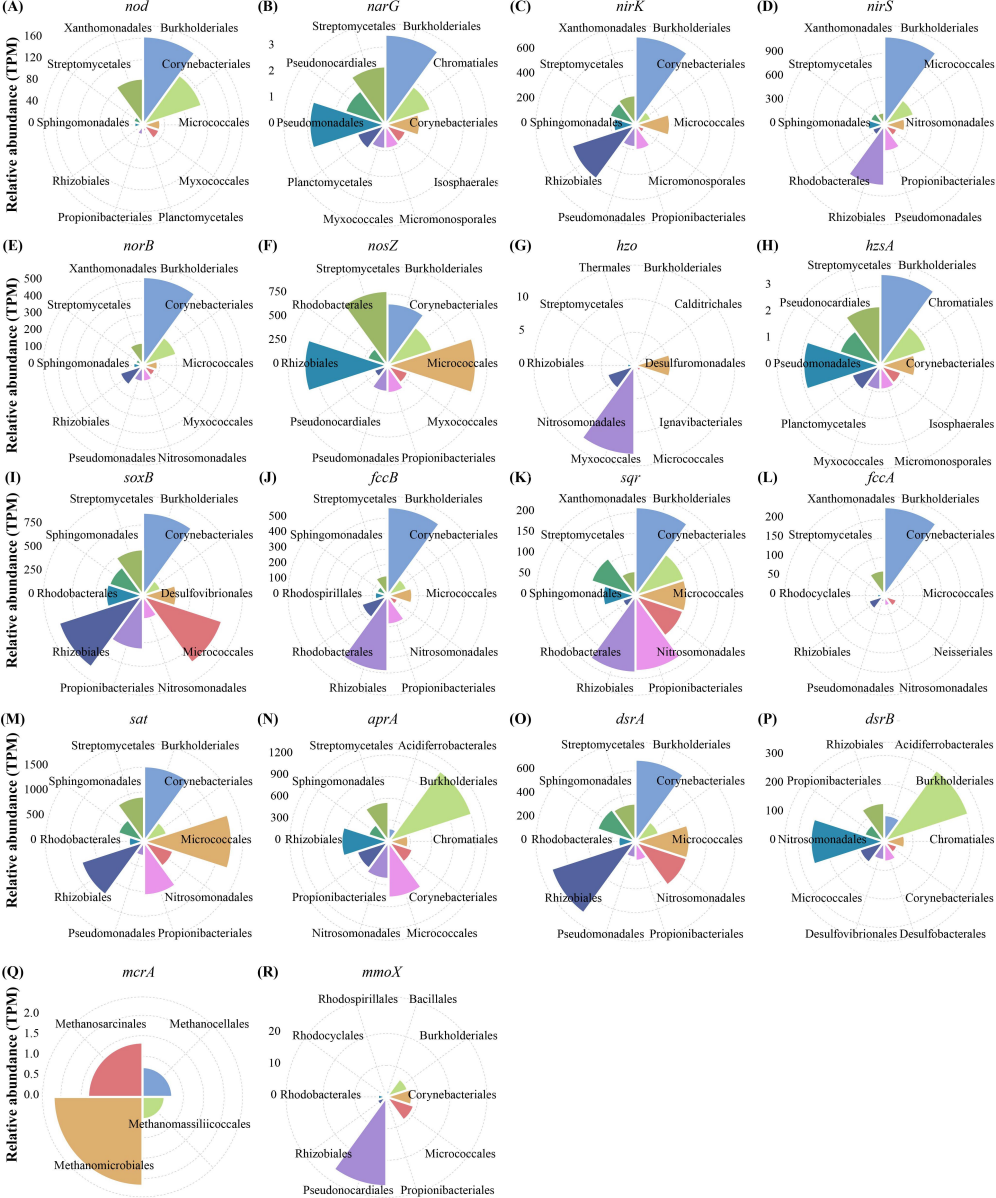
Summary of dominant microbial taxa involved in nitrogen (A through H), sulfur (I through P), and methane (Q through R) cycling. The relative abundance (TPM) for the top 10 abundant microbes (orders) involved in each gene family is shown in the wind rose diagrams. The *mcrA* was only affiliated with four orders (Methanosarcinales, Methanocellales, Methanomassiliicoccales, and Methanomicrobiales).

We assessed the relative abundance of functional genes associated with both methanogenesis and methane oxidation. For methanogenesis, three complete metabolic pathways were identified, including aceticlastic, hydrogenotrophic, and methylotrophic methanogenesis. Furthermore, the relative abundance of central methanogenic pathway (43.64%–54.29%) and aceticlastic methanogenesis (35.09%–45.63%) dominated the methanogenesis, indicating that methane production in Cuochuolong Wetland was primarily performed by acetoclastic methanogens (Fig. S3). Besides, the relative abundance of the methyl coenzyme M reductase (*mcrA*) gene in sediment was notably higher than that in other habitats (Fig. 2). The predominant taxa associated with methanogenesis were identified as Methanosarcinales, Methanocellales, Methanomassiliicoccales, and Methanomicrobiales (Fig. 3Q).

The potential of methane oxidation was significantly (*P* < 0.05) lower in sediment than that in rhizosphere and bulk soils (Fig. S4). For both rhizosphere and bulk soils, the relative abundance of aerobic methane oxidation pathway was significantly (*P* < 0.05) higher in surface layer compared with those in subsurface layer, indicating that the surface soils (R_surface_ and B_surface_) exhibited higher potential of aerobic methane oxidation than that in the subsurface soils (R_subsurface_ and B_subsurface_). The methane monooxygenase subunit A gene (*pmoA*), the marker gene for methane oxidation, which oxidizes methane to methanol, was rarely detected (Fig. 2). However, another gene that can also oxidize methane to methanol (*mmoX*) was found more abundant. The *mmoX* gene was largely affiliated with Pseudonocardiales, Micrococcales, and Corynebacteriales (Fig. 3R).

#### Nitrogen cycling

The metagenomic contigs related to nitrogen cycling were primarily associated with nitrate reduction, with 47.33%–52.64% involved in organic degradation and synthesis, 16.82%–19.62% in dissimilatory nitrate reduction (DNRA), 15.92%–18.95% in denitrification, and 10.56%–13.43% in assimilatory nitrate reduction (ANRA) (Fig. S5). Gene families involved in denitrification (*narG*, *nirK*, *nirS*, and *norB*) were increased with decreasing salinity levels across the five habitats, following the trend: sediment <R_surface_ < R_subsurface_ <B_surface_ < B_subsurface_, demonstrating negative correlations with salinity levels (Fig. 2). The major denitrifying taxa included Burkholderiales, Rhizobiales, Micrococcales, Pseudomonadales, and Xanthomonadales (Fig. 3B through E). For anammox genes, the detected *hzo* gene was mainly affiliated with Myxococcales, Desulfuromonadales, and Nitrosomonadales, and its relative abundance increased with decreasing salinity levels, whereas *hzsA* gene largely originating from Burkholderiales, Pseudomonadales, and Streptomycetales was detected with a reverse trend (Fig. 2 and Fig. 3G and H).

#### Sulfur cycling

For the sulfur cycling, the metagenomic contigs were mainly mapped to organic sulfur transformation (30.45%–33.09%), linkages between inorganic and organic sulfur transformation (20.00%–20.77%), and assimilatory sulfate reduction (17.36%–18.36%) (Fig. S6). The relative abundance of *dsrAB*, which were considered as the marker genes of dissimilatory sulfate reduction, increased along the decreasing salinity levels (Fig. 2). The detected *dsrAB* were primarily originated from Rhizobiales, Burkholderiales, and Nitrosomonadales (Fig. 3O and P). The key sulfur oxidation genes (*soxB*, *fccB,* and *sqr*) were mainly originated from Burkholderiales, Rhizobiales, Micrococcales, Propionibacteriales, and Streptomycetales (Fig. 3I through K).

### Taxonomic and functional compositions of the microbial communities in the five habitats

**Fig 4:**
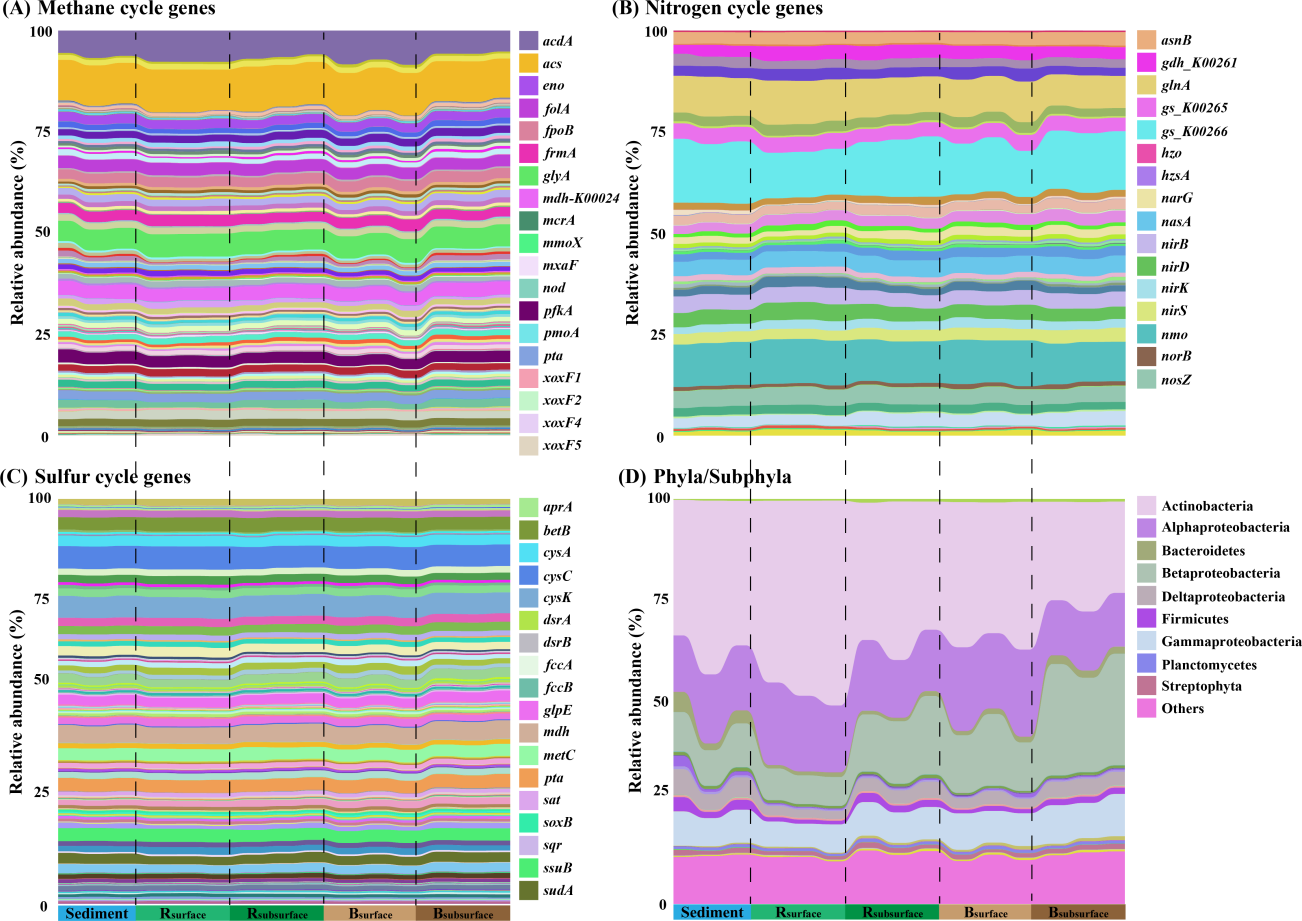
The relative abundance of methane cycle genes (**A**), nitrogen cycle genes (**B**), sulfur cycle genes (**C**), and phyla/subphyla (**D**) in different habitats and layers, derived from metagenomic sequencing data. The legend displays only the top 10 genes/phyla with the highest relative abundance and key genes of elemental cycles. Detailed information can be seen in Table S1–Table S4 (Supplementary material 2). Sediment: sediment samples; R_surface_, surface samples of the rhizosphere soils; R_subsurface_, subsurface samples of the rhizosphere soils; B_surface_, surface samples of the bulk soils; B_subsurface_, subsurface samples of the bulk soils.

**Fig 5:**
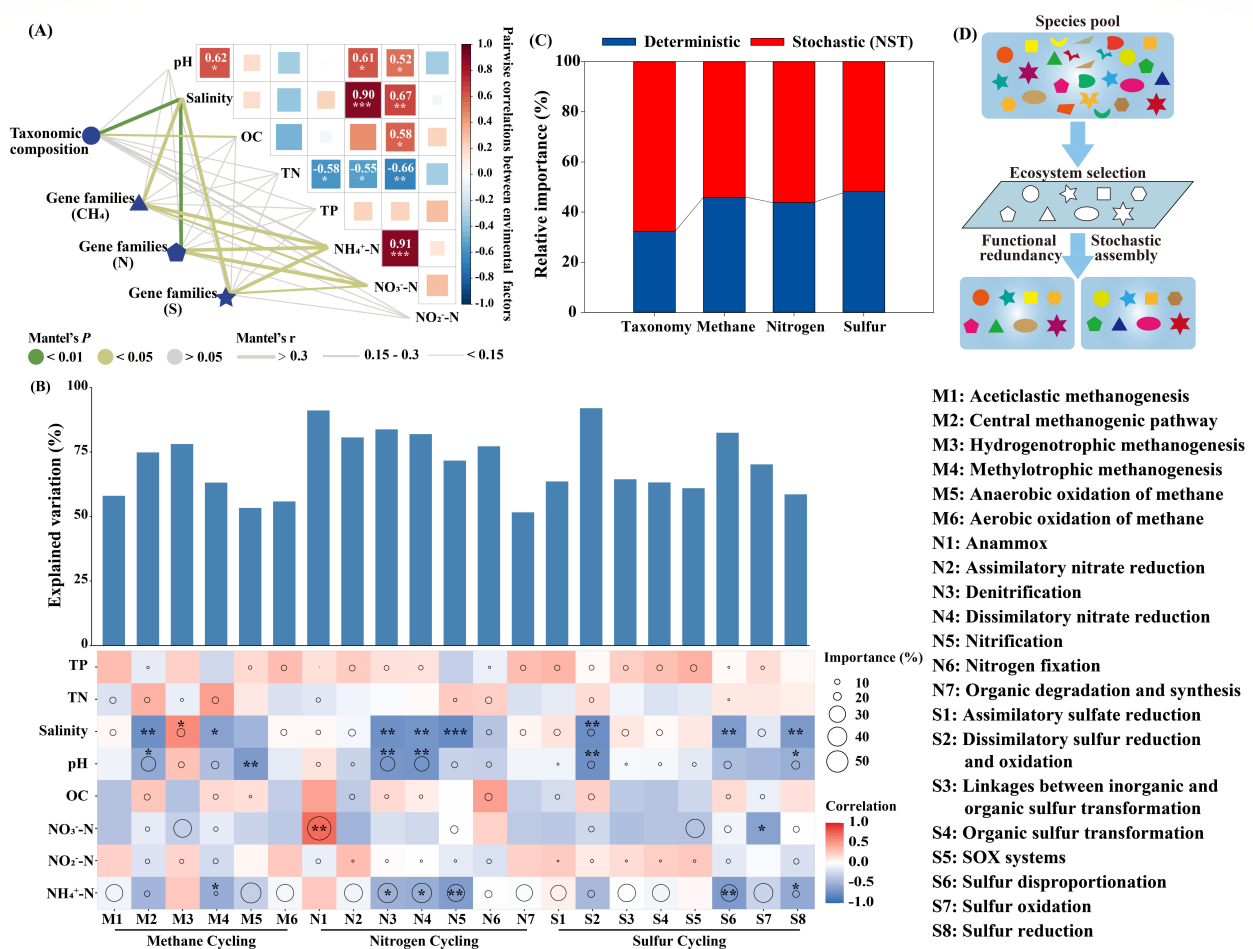
(**A**) Pairwise correlation analysis between physicochemical properties and compositions of taxonomy and functional gene families based on the Mantel tests. Spearman’s correlations were also calculated between environmental factors. The color gradient of the lines denotes the significance (Mantel’s *P*) based on 999 permutations, with the width of the lines representing correlation coefficients (Mantel’s r). Color gradient and rectangle size indicate Spearman’s correlation coefficients, and asterisks in the rectangle denote different significance levels at *P < 0.05; **P < 0.01; ***P < 0.001. (**B**) Variations explainable by different environmental factors at functional pathway level based on correlation and best multiple regression model. Circle size represents the variable importance (that is, proportion of explained variability calculated via multiple regression modeling and variance decomposition analysis). Colors and asterisk represent Spearman correlations: *P < 0.05; **P < 0.01. OC, organic carbon; TN, total nitrogen; TP, total phosphorus; NH_4_^+^-N, ammonia nitrogen; NO_3_^-^-N, nitrate nitrogen; NO_2_^-^-N, nitrite nitrogen. (**C**) The relative importance of deterministic and stochastic processes in taxonomic and functional compositions of microbial community based on the Null model. (**D**) A conceptual model for formation mechanism of microbial community. First, a regional species was formed, adapting to ecological niches in the saline-alkaline wetland of the Tibetan Plateau. Second, the ecosystem selects microbial functions rather than species, unless they are highly specialized. Third, functional redundancy of microbial species leads to stochastic taxonomic community composition. In this model, different shapes represent distinct functions, while varying colors indicate different species.

In each habitat, the relative abundance of methane, nitrogen, and sulfur cycle genes was relatively even across samples, though a few exceptions were observed (Fig. 4A through C; Tables S1 to S3). Conversely, their taxonomic compositions varied dramatically (Fig. 4D; Table S4), even at the phylum level, indicating clear functional redundancy. This pattern was also observed across the five habitats, suggesting functional convergence. Similar patterns were also observed in the results derived from the 16S rRNA gene data, further confirming the widespread presence of functional redundancy across the five habitats (Fig. S7). Moreover, the functional redundancy index (FRI) of each KEGG Orthology (KO) provided additional evidence of the presence of functional redundancy (Fig. S8; Table S7). A total of 8,521 KOs were predicted across the five habitats based on the functional prediction from the 16S rRNA gene data (Table S7). Among them, 8,428, 8,400, 8,368, 8,380, and 8,381 KOs exhibited functional redundancy (FRI >0) in sediment, R_surface_, R_subsurface_, B_surface_, and B_subsurface_, respectively (Fig. S8; Table S7). Results derived from both metagenomic sequencing and 16S rRNA gene data revealed distinct patterns in the taxonomic and functional compositions among the five habitats (Fig. S9 and S10). To disentangle potential environmental drivers of microbial compositions, Mantel tests were performed. The results revealed that salinity was the most dominant factor shaping both the taxonomic and functional compositions (Fig. 5A). Specifically, NH_4_^+^-N and NO_3_^-^-N only have a significant influence on functional compositions, but not on taxonomic composition (*P* < 0.05). Environmental factors influencing the microbial functional compositions were also strongly associated with individual functional pathways (Fig. 5B). Almost all key pathways of methane, nitrogen, and sulfur cycling were influenced by salinity, pH, and NH_4_^+^-N. For example, salinity negatively influenced the central methanogenic pathways, methylotrophic methanogenesis, denitrification, dissimilatory nitrate reduction, nitrification, dissimilatory sulfur reduction and oxidation, sulfur disproportionation, and sulfur reduction. NH_4_^+^-N influenced methylotrophic methanogenesis, denitrification, dissimilatory nitrate reduction, nitrification, sulfur disproportionation, and sulfur reduction. However, the relationship between environmental factors and individual microbial taxa was limited (Fig. S11). Only three dominant phyla (Actinobacteria, Betaproteobacteria, and Gammaproteobacteria) were significantly correlated with salinity (Fig. S11). Null model analysis was subsequently utilized to evaluate the relative importance of deterministic and stochastic processes in shaping the microbial community. Consistent with our hypothesis, taxonomic structures were less influenced by deterministic processes compared to functional compositions, with deterministic processes contributing 32.39% to taxonomic compositional variations (Fig. 5C). The relative importance of deterministic processes in methane, nitrogen, and sulfur functional compositions reached 45.78%, 43.79%, and 48.19%, respectively. The similar patterns were observed at individual habitats (Fig. S12). Furthermore, the relative importance of stochastic processes increased as salinity levels decreased across the five habitats, following the trend: sediment <R_surface_ < R_subsurface_ <B_surface_ < B_subsurface_ (Fig. S12), that is, as salinity levels increased, the relative importance of deterministic processes also increased. Null model analysis based on the 16S rRNA gene data also revealed that deterministic processes have a greater influence on functional compositions than on taxonomic structures (Fig. S13). The contribution of stochastic processes also increased with decreasing salinity across the five habitats, following the trend: sediment <R_surface_ < R_subsurface_ <B_surface_ < B_subsurface_ (Fig. S13), providing further evidence that deterministic processes were more dominant under higher salinity conditions. Despite habitat heterogeneity, the alpha diversity of microbial communities exhibited limited variation across the five habitats (Fig. S14).

### Genomic potential of methane, nitrogen, and sulfur cycling processes

To further characterize the microbes and their genetic mechanisms associated with methane, nitrogen, and sulfur cycling, a total of 188 non-redundant medium- and high-quality MAGs were reconstructed through the metagenomic binning of contigs (Fig. S15; Table S8). Among them, 187 MAGs were classified as bacteria, and the remaining one was archaea. Among the bacterial MAGs, 73 sulfur-driven denitrifier MAGs containing key genes for sulfur oxidation and denitrification were identified (Table S9). These sulfur-driven denitrifier MAGs were distributed across Acidobacteriota, Actinobacteriota, Bacteroidota, Campylobacterota, Chloroflexota, Desulfobacterota, Desulfobacterota_E, Firmicutes, Gemmatimonadota, Krumholzibacteriota, Planctomycetota, Proteobacteria, and Verrucomicrobiota (Table S9).

**Fig 6:**
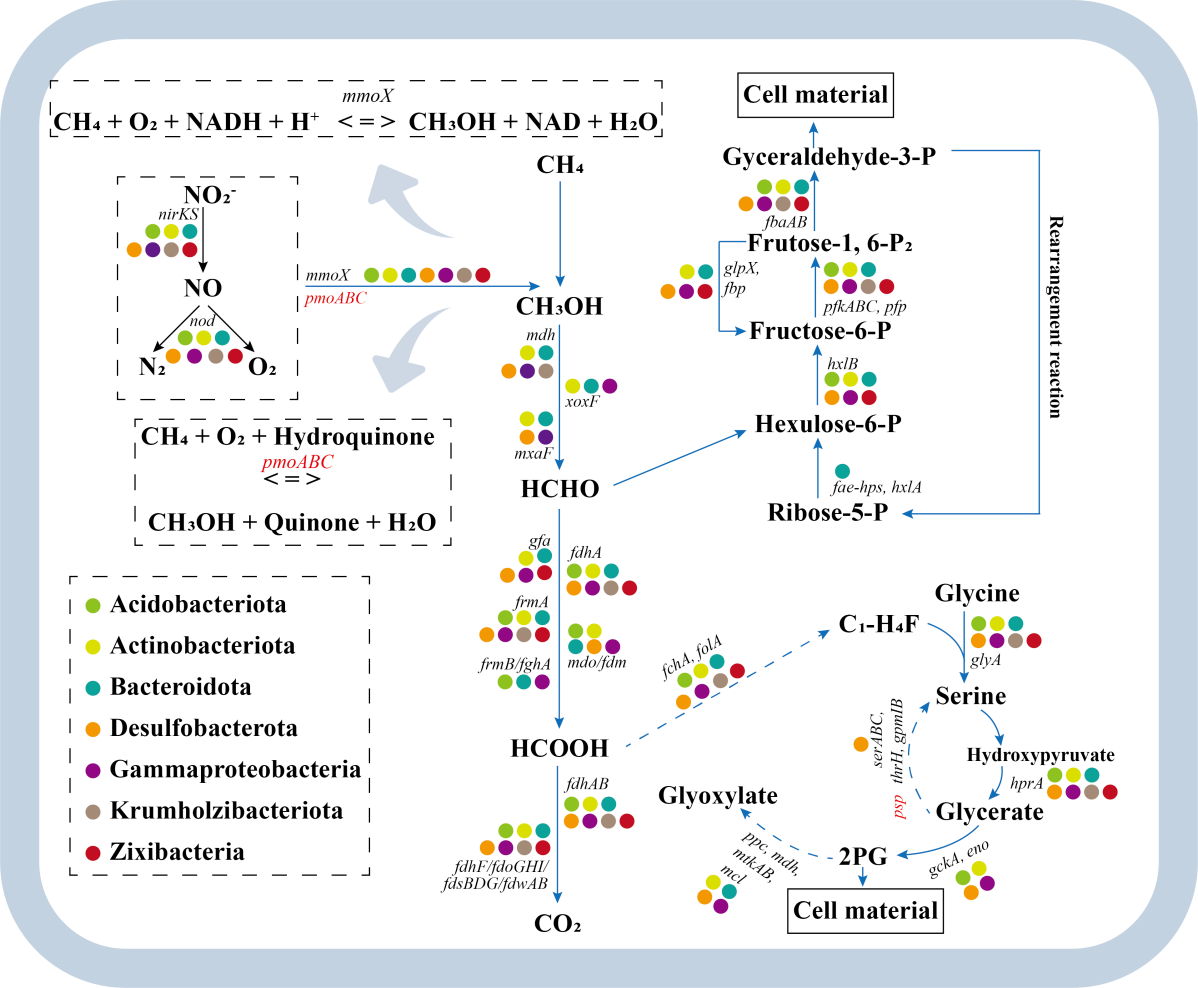
Predicted pathways of methane oxidation in the seven phyla/subphyla, based on the analysis of 18 bins. Acidobacteriota, bin30; Actinobacteriota, bin1, bin7, bin15, and bin76, Bacteroidota: bin111, bin119, bin128, bin131, and bin167; Desulfobacterota, bin27 and bin145; Gammaproteobacteria, bin9, bin35, bin123, and bin163; Krumholzibacteriota, bin164; Zixibacteria, bin82).

Furthermore, we identified 18 MAGs that contained the *nod* gene, while none of them contained the *pmoA* gene (Fig. 6). Besides, all 18 MAGs did contain the *mmoC* or *mmoX* gene. These 18 MAGs were distributed across Acidobacteriota (bin30), Actinobacteriota (bin1, bin7, bin15, and bin76), Bacteroidota (bin111, bin119, bin128, bin131, and bin167), Desulfobacterota (bin27 and bin145), Gammaproteobacteria (bin9, bin 35, bin123, and bin163), Krumholzibacteriota (bin164), and Zixibacteria (bin82) (Table S10). At the genus level, 13 of these 18 MAGs were assigned to identifiable genera, including WHSW01 (bin1), XN24 (bin9), Desulforhopalus (bin27), 2-02-FULL-61-13 (bin35), UBA4719 (bin76), Draconibacterium (bin111), UBA6688 (bin119), Ramlibacter (bin123), IGN2 (bin128), Algoriphagus (bin131), JABZFP01 (bin145), GCA-2722315 (bin163), and Glo-17 (bin164). Notably, five MAGs (bin7, bin15, bin30, bin82, and bin167) could not be classified to a known genus. Only one MAG (bin119) was assigned to the species level, identified as UBA6688 sp002454845, while the remaining 17 MAGs could not be annotated at the species level, suggesting the presence of potentially novel taxa (Table S10). A notable divergence in the relative abundance of these 18 MAGs among the five habitats was observed (Fig. S16). Additionally, they contained almost all key gene families for methane oxidation (Fig. 6).

Subsequently, to assess the generalizability of our results, 50 genomes of Acidobacteriota (genome1–genome5), Actinobacteriota (genome6–genome11), Bacteroidota (genome12–genome24), Desulfobacterota (genome25–genome26), Gammaproteobacteria (genome27–genome39), Krumholzibacteriota (genome40–genome43), and Zixibacteria (genome44–genome50) were downloaded from NCBI (Table S11). All these genomes contained the *nod* and *mmoX* genes, except for genome21 (Bacteroidota), genome41 (Krumholzibacteriota), and genome49 (Zixibacteria), which did not contain the *mmoX* genes.

**Fig 7:**
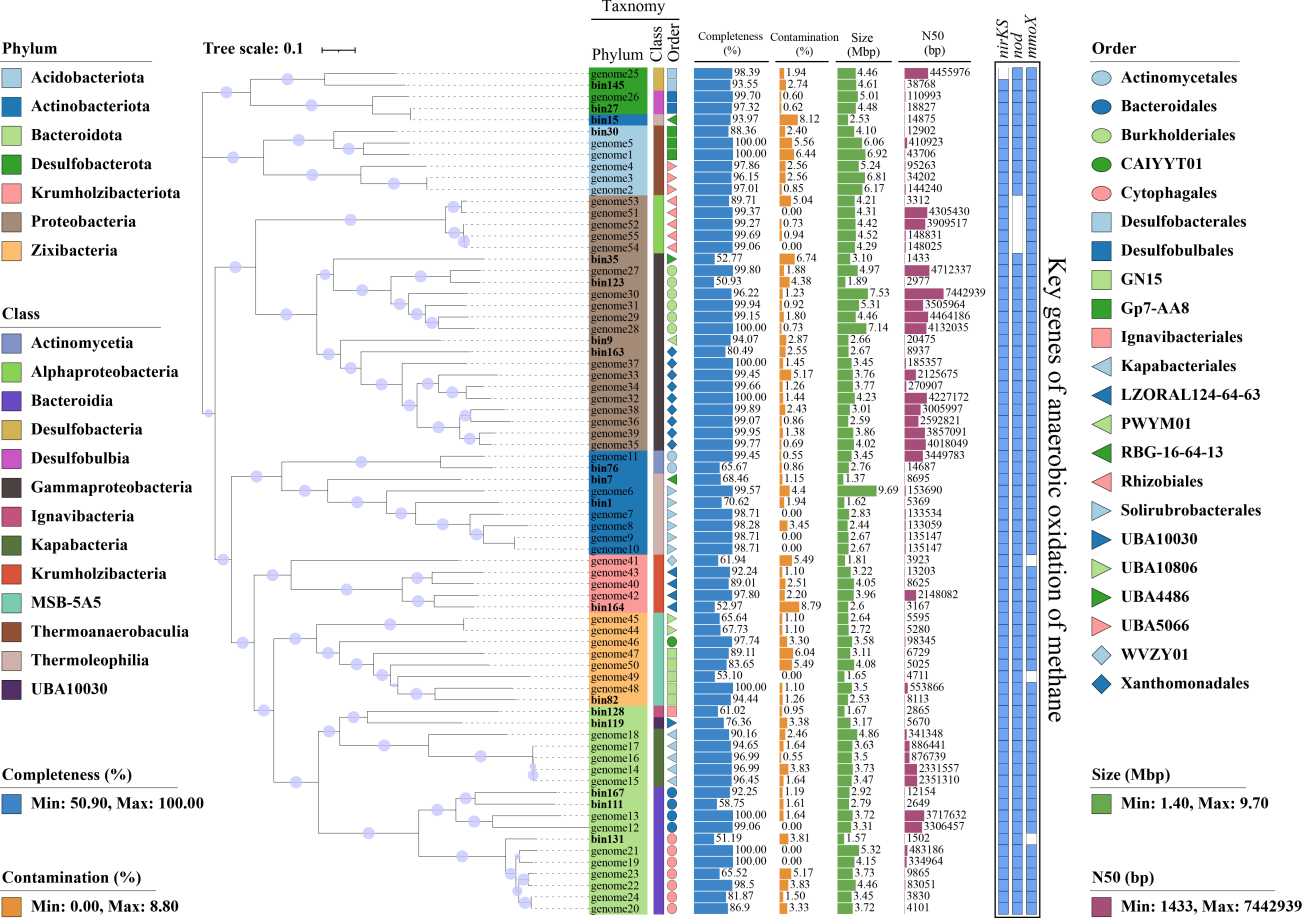
Phylogenetic tree of Acidobacteriota, Actinobacteriota, Bacteroidota, Desulfobacterota, Gammaproteobacteria, Krumholzibacteriota, and Zixibacteria comprising 55 genomes downloaded from National Center for Biotechnology Information (NCBI, https://www.ncbi.nlm.nih.gov/) and European Molecular Biology Laboratory-European Bioinformatics Institute (EMBL-EBI, https://www.ebi.ac.uk) and 18 metagenomic assembled genomes (MAGs) reconstructed through metagenomic binning and the presence-absence profile of key gene families encoding enzymes involved in methane oxidation. Names highlighted in bold represent the MAGs obtained from the present study, and the other genomes were sourced from NCBI and EMBL-EBI (See Table S11 for detailed information on these genomes). Bootstrap values were determined using non-parametric bootstrapping with 100 replicates and represented by purple circles of varying sizes. The scale bar indicated 10% estimated phylogenetic divergence. The taxonomic affiliations of each genome were differently colored according to GTDB-Tk. The presence of gene families was indicated by filled blue boxes.

Additionally, five genomes (genome51–genome55) of Methylocella, the only known genus of methanotrophs that possesses the *mmoX* gene without the *pmoA* gene, were downloaded (Table S11). As anticipated, all five genomes harbored the *mmoX* gene in the absence of the *pmoA* gene, indicating their potential for methane oxidation (Fig. 7). However, none of the genomes contained the *nod* gene, suggesting that they are not capable of nitric oxide disproportionation (Fig. 7). A phylogenetic tree comprising the 55 downloaded genomes and the 18 MAGs containing the *nod* and *mmoX* genes was constructed (Fig. 7). In the phylogenetic tree, the five genomes of Methylocella formed a separate cluster.

## DISCUSSION

Revealing the distribution of methane, nitrogen, and sulfur cycling genes/pathways and associated taxonomic groups is vital to understand the ecosystem functioning of plateau saline-alkaline wetlands (28). Our results demonstrated a notable divergence in the distribution of these functional genes and pathways among the sediment, R_surface_, R_subsurface_, B_surface_, and B_subsurface_ within the Cuochuolong Wetland.

Methane cycling is a crucial component of the carbon cycle, and its emissions significantly accelerate global warming, given its warming potential approximately 84 times greater than that of carbon dioxide over a 20-year period (42, 43). Moreover, wetlands represent the largest natural source of methane, contributing about 20%–40% of total methane emissions (44). Methanogenesis, conducted by methanogens containing the *mcrA* gene, typically occurs in anoxic habitats (45). In the present study, we observed a relatively high abundance of the *mcrA* gene in sediment, indicating that the sediment possessed a relatively higher potential for methanogenesis compared with other habitats (Fig. 2). Sediment was characterized by oxygen limitation due to its water-logged condition (46). Therefore, the sediment was more suitable for methanogens and more conducive to the methanogenic process. For methane oxidation, previous studies have indicated that this process predominantly occurs at the oxic-anoxic transition zone (47). In this study, we also found that the potential of methane oxidation was relatively higher in rhizosphere and bulk soils, which typically represent the oxic-anoxic transition zone in wetland ecosystems, compared with anoxic sediment (Fig. S4). This pattern of methane-oxidizing potential reflected oxygen-mediated habitat partitioning (48). Additionally, substrate availability and potential interactions among microbes may further contribute to the observed differences. Additionally, consistent with previous studies, the surface soils were found to exhibit a higher potential for aerobic methane oxidation compared with subsurface soils, likely due to the higher oxygen levels at the surface (28, 49).

Microbially driven nitrogen cycling is vital for maintaining nitrogen levels in such nutrients-limited ecosystems (50). Denitrification is recognized as a primary nitrogen-loss pathway within natural ecosystems, through the production of nitrous oxide and/or dinitrogen (51). Our results revealed that the functional potentials of denitrification increased with decreasing salinity levels across the five habitats (Fig. 2), following the trend: sediment <R_surface_ < R_subsurface_ <B_surface_ < B_subsurface_, suggesting that salinity acts as an inhibiting factor in the denitrification process. The results of the Spearman’s correlation and best multiple regression model further confirmed that salinity negatively influenced the denitrification pathway (Fig. 5B). Previous studies also indicated that salinity was negatively correlated with denitrification (52, 53).

Microbial sulfur metabolism plays a vital role in biogeochemical cycling, interlinked with other elemental cycles, thus bearing significant environmental implications (54, 55). The plateau saline-alkaline wetlands, characterized by high salinity, are abundant in sulfate, which may fuel efficient sulfur cycling. Sulfate reduction was considered one of the most important respiratory processes in natural ecosystems, while sulfur oxidation holds potential for detoxifying sulfide from the root zone to benefit plants (28, 56). The *dsrAB* genes were considered the marker of dissimilatory sulfate reduction (57, 58). In the present study, the relative abundance of *dsrAB* increased along the decreasing salinity levels, indicating that the zone with relatively low salinity serves as hotspots for dissimilatory sulfate reduction within plateau saline-alkaline wetlands. The results of Spearman’s correlation and best multiple regression model further confirmed that salinity negatively influenced the sulfate reduction pathways (Fig. 5B).

A long-standing challenge in microbial ecology is understanding the formation mechanism of complex communities (31). The prevailing consensus suggests that microbial communities are shaped by a combination of deterministic and stochastic processes, with their relative importance being the key question (59, 60). Recently, several studies have proposed that the environment selected for microbial function rather than species, thus making the functional compositions of microbial communities highly deterministic (18, 61, 62). However, widespread functional redundancy among microbial taxa underlies stochastic taxonomic community structure (25, 63). In the present study, we also observed potential functional redundancy among microbial taxa (Fig. 3 and 4; Fig. S7 and S8). Moreover, the functional compositions of microbial communities involved in methane, nitrogen, and sulfur cycling were more significantly influenced by deterministic processes than taxonomic structures (Fig. 5C). Both the taxonomic and functional compositions were primarily influenced by salinity (Fig. 5A). However, environmental factors were more important in shaping the functional composition of microbial communities than taxonomic composition (Fig. 5A and B; Fig. S11). These results supported the hypothesis that the extreme environment of plateau saline-alkaline wetlands, particularly salinity, selected for microbial function rather than species, with functional redundancy underpinning stochastic taxonomic community compositions. Similar patterns were observed within individual habitats, with the relative importance of deterministic processes increasing as salinity levels increased across the five habitats (Fig. S12). Consistent with our findings, an increase in salinity was also found to enhance deterministic processes in high-salinity lakes (64). In summary, we further proposed a hypothesized model to elucidate the formation mechanism of microbial communities (Fig. 5D). First, distinct habitats (e.g., sediment, rhizosphere soils, and bulk soils) are generated in the plateau saline-alkaline wetland by various physicochemical characteristics, such as salinity, oxygen, and depth. Microbes capable of surviving in these habitats constitute the regional species pools (65). Second, the environment of distinct habitats selects for microbial function rather than species, unless the microbial species exhibit exceptional specialization in specific functions, such as anaerobic methane oxidation (61). Specifically, the environment acts as a shape sorter that contains different shape filters. These shape filters deterministically select microbes from nearby species pools according to their functions. Microbial species with selected shape (functions), despite their color (species), have equal possibility to pass the shape filter (66). That is, microbial species with functions that match the environmental functional filters, irrespective of their taxa, can be selected stochastically coinciding with the neutral theory (5). Third, these randomly selected successful microbes outcompete other microbes, colonize the environment, and occupy the niche. Consequently, diverse microbial species with similar functions can randomly occupy the same niche in an ecosystem, leading to the phenomenon known as microbial functional redundancy (25, 67). Additionally, functional redundancy theoretically enhances community stability by buffering against environmental perturbations through compensatory mechanisms (25).

In the present study, the five habitats selected similar microbial functions, including methane, nitrogen, and sulfur cycling, suggesting functional convergence (68). This phenomenon resembles the observed convergence in community function despite taxonomic divergence, as seen in other extreme environments, such as anaerobic bioreactors and deep ocean sediments (24, 69). George et al. (70) developed a microbial community consumer-resource model that provided a possible explanation for the microbial functional convergence observed in many extreme environments. They demonstrated that in harsh environments, the thermodynamics of microbial growth led to functional convergence (70). In our study, all five habitats were located within Cuochuolong wetland, a typical saline-alkaline wetland characterized by high altitude and high salinity. Consequently, these five similar harsh habitats may display microbial functional convergence. Further investigation into microbial functional convergence, such as a comparison of microbial functions between regular and harsh environments, is reserved for future work.

The nitric oxide dismutase was presumed to catalyze the disproportionation of nitric oxide into dinitrogen and oxygen, constituting a unique link between the carbon and nitrogen cycles (71). This dismutase was encoded by the *nod* gene, which was initially thought to be present only in anaerobic methane-oxidizing bacteria within the NC10 phylum (Candidatus *Methylomirabilis oxyfera*) (72). Recent studies have expanded the diversity of microbes containing the *nod* gene, including Alphaproteobacteria, Gammaproteobacteria, and Planctomycetia, suggesting their potential involvement in the disproportionation of nitric oxide (37). In the present study, we also observed 18 MAGs affiliated with Acidobacteriota, Actinobacteriota, Bacteroidota, Desulfobacterota, Gammaproteobacteria, Krumholzibacteriota, and Zixibacteria, each containing the *nod* gene (Fig. 6). We then downloaded 50 genomes affiliated with these seven phyla from the NCBI to assess the generalizability of our finding. The annotation results confirmed the presence of the *nod* gene in all samples (Fig. 7), indicating the potential involvement of these seven phyla in the disproportionation of nitric oxide into dinitrogen and oxygen. Previous studies overlooked this finding, maybe because the earlier databases did not reflect the *nod* gene as a biogeochemical cycling gene, despite its recent recognition as a potential contributor to nitrogen and methane cycling (36). However, the annotation of MAGs heavily relies on the coverage and accuracy of databases (43). The development of manually curated databases, such as MCycDB and NCycDB, which provide high specificity, coverage, and accuracy, allows for more comprehensive and accurate profiling of biogeochemical cycling microbial communities (43, 73). Moreover, plateau saline-alkaline wetlands feature a dynamic oxic-anoxic interface coupled with low concentrations of oxidizable substrates, which theoretically facilitate nitric oxide dismutation. Therefore, microbes involved in the disproportionation of nitric oxide were expected to colonize in plateau saline-alkaline wetlands. Additionally, five of these MAGs could not be classified into a known genus, while 17 MAGs could not be annotated at the species level, suggesting that these MAGs may represent potentially novel taxa. This finding may further expand our knowledge of the diversity of microbes containing the *nod* gene, implying that more taxa could potentially contribute to nitrogen loss. Nevertheless, targeted experimental validation is essential to definitively confirm the presence of *nod* genes in these microbial groups.

Furthermore, the oxygen released during the disproportionation of nitric oxide was suggested to be involved in nitrite-dependent anaerobic methane oxidation (n-DAMO) by the NC10 phylum (40). Theoretically, in this pathway, methane is oxidized into methanol and water catalyzed by the enzyme methane mono-oxygenase (MMO) (34). The NC10 phylum bacteria contained the *pmoA* gene encoding the particulate (membrane-bound) form of this enzyme (pMMO); *mmoX* gene encoding the soluble form was absent (34). The 18 MAGs observed in our study (Acidobacteriota, Actinobacteriota, Bacteroidota, Desulfobacterota, Gammaproteobacteria, Krumholzibacteriota, and Zixibacteria), which contain the *nod* gene, did not contain the *pmoA* gene but did contain the *mmoX* gene (Fig. 6). The *mmoX* gene encodes the soluble form of MMO, which can also catalyze the oxidation of methane into methanol and water (74, 75). Additionally, these 18 MAGs contained almost all key genes for methane oxidation (Fig. 6). The 50 genomes downloaded from the NCBI also contained the *nod* and *mmoX* genes, with the exceptions of genome21 (Bacteroidota), genome41 (Krumholzibacteriota), and genome49 (Zixibacteria), which lacked the *mmoX* genes. Based on these findings, we hypothesized that the 18 MAGs (Acidobacteriota, Actinobacteriota, Bacteroidota, Desulfobacterota, Gammaproteobacteria, Krumholzibacteriota, and Zixibacteria), which contain the *nod* and *mmoX* genes, were potentially involved in n-DAMO despite lacking the *pmoA* gene. Consistent with our hypothesis, analysis of Methylocella genomes, a genus of methanotrophs, indicated that they contain the *mmoX* gene but lack the *pmoA* gene (76). The results of quantitative real-time PCR, growth experiments, and cloning of 16S rRNA genes and fluorescence *in situ* hybridization further confirmed that Methylocella utilizes only the sMMO, encoded by the *mmoX* gene, to catalyze methane oxidation and lacks the *pmoA* gene, which encodes the pMMO (77, 78). Therefore, we further hypothesize that the 18 MAGs obtained from our study may oxidize methane to methanol via the sMMO encoded by the *mmoX* gene, similar to Methylocella. After the initial oxidation of methane to methanol, methanol dehydrogenase oxidizes methanol to formaldehyde, which can be assimilated into cell carbon or further oxidized to formate and CO_2_ for energy generation (79). This revealed an unexpected predicted methane metabolism, suggesting that these taxa were a previously overlooked microbial methane sink, although further investigation is required.

As Methylocella is the only known genus of methanotrophs containing the *mmoX* gene without the *pmoA* gene (76, 77). We also downloaded five Methylocella genomes and examined them for the presence of *nod*, *mmoX*, and *pmoA* genes. As expected, all five genomes contained the *mmoX* gene without the *pmoA* gene (Fig. 7), indicating their potential for methane oxidation. However, none of these genomes contained the *nod* gene, suggesting that they are not capable of nitric oxide disproportionation. Additionally, these five genomes form a separate cluster in the phylogenetic tree (Fig. 7). Based on these findings, we hypothesized that Methylocella and the 18 MAGs obtained from our study may oxidize methane through a similar pathway; however, the sources of oxygen may differ (80). The 18 MAGs containing the *nod* gene may generate oxygen via the dismutation of nitric oxide into dinitrogen and oxygen. In contrast, Methylocella, which lacks the ability to perform nitric oxide disproportionation, likely relies on environmental oxygen (81). However, to date, direct evidence supporting the involvement of these taxa in methane oxidation remains lacking. We will verify this finding by isolated culture, stable isotope measurements (^14^CH_4_), metatranscriptomic sequencing, and methane monooxygenase gene clone library analyses in our upcoming study to directly detect methane oxidation activity in these taxa.

### Conclusions

Our metagenomic sequencing analysis unveiled the distribution of methane, nitrogen, and sulfur cycling microbial communities and their formation mechanism within the plateau saline-alkaline wetland. The results indicated a notable divergence in the distribution of methane, nitrogen, and sulfur cycling pathways among the sediment, R_surface_, R_subsurface_, B_surface_, and B_subsurface_ within the Cuochuolong Wetland. The sediment had relatively higher functional potentials for methanogenesis but lower functional potentials for methane oxidation. Furthermore, the functional potentials of denitrification and dissimilatory sulfate reduction increased with decreasing salinity levels across the five habitats, following the trend: sediment <R_surface_ < R_subsurface_ <B_surface_ < B_subsurface_. In each habitat, the taxonomic compositions of microbial communities exhibited high variability across samples, whereas functional genes associated with methane, nitrogen, and sulfur cycling demonstrated a relatively even distribution, indicating clear functional redundancy properties. The functional compositions of microbial communities involved in methane, nitrogen, and sulfur cycling were more significantly influenced by deterministic processes than taxonomic structures, as revealed by Null model analysis. Furthermore, salinity was the most dominant factor shaping both the taxonomic and functional compositions. These results further confirmed that the extreme environment of the plateau saline-alkaline wetland, particularly salinity, deterministically selected for microbial functions rather than species, with functional redundancy underpinning stochastic taxonomic community compositions. Moreover, we reconstructed 188 non-redundant medium- and high-quality MAGs, with 18 MAGs across seven phyla—including Acidobacteriota, Actinobacteriota, Bacteroidota, Desulfobacterota, Gammaproteobacteria, Krumholzibacteriota, and Zixibacteria—that contain the *nod* gene. These taxa could potentially be involved in the disproportionation of nitric oxide and exhibit potential for n-DAMO processes, although further investigation remains required. Overall, this study provided a comprehensive perspective of the distribution of methane, nitrogen, and sulfur cycling microbial communities and enhanced our understanding of formation mechanism of microbial community within the plateau saline-alkaline wetlands. Additionally, the present study provides evidence supporting an essential microbial ecological theory—the extreme environment of plateau saline-alkaline wetlands, particularly salinity, deterministically selected for microbial function rather than species; functional redundancy underlies stochastic taxonomic community compositions. Furthermore, this study enhances our understanding of the diversity of microbes containing the *nod* gene, which may substantially contribute to global methane and nitrogen budgets.

## MATERIALS AND METHODS

### Study site and sample collection

Cuochuolong Wetland (29°6′5.73″N, 85°23′30.21″E) is a saline-alkaline wetland located in the Tibetan Plateau, with an average elevation exceeding 4,600 m a.s.l (41). In August 2020, sediment, rhizosphere soil, and bulk soil samples were collected in the Cuochuolong Wetland, where *Puccinellia himalaica* thrives as the dominant macrophyte. All samples were collected via Kajak soil corer (KC Denmark A/S, Holmbladsvej, Silkeborg, Denmark). Rhizosphere soils were sampled from the root zone of the predominant plant, following our previous research (27), by shaking off loosely adhering soil from the roots. Bulk soils without any root tissue were sampled at least 1 m away from vegetation at each sampling site. For rhizosphere and bulk soils, each sample was divided into surface samples (0–4 cm) and subsurface samples (4–8 cm). In total, samples were collected from five habitats, i.e., sediment, surface of rhizosphere soils (R_surface_), subsurface of rhizosphere soils (R_subsurface_), surface of bulk soils (B_surface_), and subsurface of bulk soils (B_subsurface_). Three replicate samples were collected for each habitat; hence, a total of 15 samples were obtained. The samples were stored in a car refrigerator, and then promptly transported to the laboratory. Subsequently, all samples were prepared and dried using a freeze dryer (Labconco FreeZone 4.5). After that, they were ground, homogenized, and preserved at low temperature. The samples utilized for measuring the physicochemical characteristics were stored at 4°C, while those used for DNA extraction were kept at −70°C.

### Physicochemical characteristics of sediment, rhizosphere soils, and bulk soils

Physicochemical characteristics of each sample, including pH, organic carbon (OC), total nitrogen (TN), total phosphorus (TP), ammonia nitrogen (NH_4_^+^-N), nitrate nitrogen (NO_3_^-^-N), and nitrite nitrogen (NO_2_^-^-N) were measured according to standard methods as previously described (27, 82). The salinity was measured using 2.0 g of dry sample in a 1:5 sample/water suspension with a multi-parameter water quality analyzer (Leici, DZB-718L, Shanghai, China), following Qian et al. (28).

### DNA extraction, sequencing, and sequence processing

The DNA extraction and sequencing were constructed at Guangdong Magigene Biotechnology Co. Ltd. In brief, the microbial DNA of each sample was extracted from a 0.25 g dried sample (soil or sediment) using the E.Z.N.A. stool DNA Kit (Omega Bio-tek, Norcross, GA, U.S.) according to the manufacturer’s protocols. The quality of each DNA sample was assessed using a BioPhotometer (Eppendorf, Hamburg, Germany).

For shotgun metagenomic sequencing, genomic DNA (1 µg) of each sample was fragmented by the Covaris S220 Focused-ultrasonicator (Woburn, MA USA), and sequencing libraries were prepared with a fragment length of 450 bp. Sequencing was conducted on the Illumina HiSeq X instrument, employing pair-end 150 bp mode, resulting in the generation of 2 × 150 bp paired-end reads with an average of 10 Gb per sample. All sequenced data were submitted to the National Omics Data Encyclopedia (NODE) under the project number OEP004316 (experiment ID OEX024507).

The metagenomic sequencing data were analyzed according to the standard pipeline (83, 84). The binning analysis was performed via the metaWRAP (v1.2.3) (85). Details of metagenomic sequencing and genome binning analysis were shown in the Supplementary material1.

For 16S rRNA gene sequencing, the primer pair 515F (5′-GTGYCAGCMGCCGCGGTAA-3′) and 806R (5′-GGACTACNVGGGTWTCTAAT-3′) was used to amplify the V4 hypervariable regions of the microbial 16S rRNA gene. The PCR system preparation and thermal cycling conditions for PCR amplification were performed as described in our previous study (86). Each DNA sample was amplified individually in triplicate, and the resulting products were combined and purified using the PowerClean DNA gel purification kit (MoBio Laboratories, Carlsbad, California, USA). Sequencing was conducted on an Illumina HiSeq-PE250 platform. The raw sequencing data have been uploaded to the National Omics Data Encyclopedia (NODE) under the project number OEP00002737 (experiment ID OEX00014566).

The amplicon sequencing data were processed using QIIME2, as outlined in our previous study (87). Details of amplicon sequence processing were provided in the Supplementary Information1.

### Statistical analysis

Nonmetric multidimensional scaling (NMDS) plots of the taxonomic-based and functional-based Bray-Curtis dissimilarities of microbial community compositions were generated via the “*vegan*” package in R (88, 89). The Mantel test (9,999 permutations) was utilized to evaluate the correlations between microbial community compositions (both taxonomic-based and functional gene-based) and environmental parameters (90). The Spearman’s correlations between environmental parameters were evaluated using SPSS (v22.0) (91). Significant differences in the concentration of physicochemical characteristics and the relative abundance of key functional genes/pathways (methane, nitrogen, and sulfur cycle) among the five habitats were tested via the one-way ANOVA in SPSS (v22.0) (92). The relative importance of deterministic and stochastic processes in structuring the microbial community composition was determined via the Null model (6). Functional prediction and FRI calculation based on the 16S rRNA gene data were performed using the “*Tax4Fun2*” package in R (93). The variations explainable by environmental properties at the individual pathway level were determined by the multiple regression model with variance decomposition analysis using the “*psych*” (94), “*reshape2*” (95), “*relaimpo*” (96), “*MASS*” (97) packages in R (31). Heatmaps, wind rose diagrams, and alluvial diagrams were visualized via the “*pheatmap*” (98), “*ggplot2*” (99), and “*ggalluvial*” (100) package in R, respectively.

## SUPPLEMENTAL MATERIAL

Supplemental file 1 aem.02206-24-s0001.docx Supplemental methods and Fig. S1 to S16.

Supplemental file 2 aem.02206-24-s0002.xlsx Tables S1 to S11.

ASM does not own the copyrights to Supplemental Material that may be linked to, or accessed through, an article. The authors have granted ASM a non-exclusive, world-wide license to publish the Supplemental Material files. Please contact the corresponding author directly for reuse.
