# Supplementary material for: Environment selected microbial function rather than taxonomic species in a plateau saline-alkaline wetland
Source: Appl Environ Microbiol. 2025 Jul 3;91(7):e02206-24. doi: 10.1128/aem.02206-24 (PMC12285268; doi:10.1128/aem.02206-24)
Supplement: Supplemental file 1 — Supplemental methods and Fig. S1 to S16. [file aem.02206-24-s0001.docx]

**SUPPLEMENTARY MATERIAL1**

**Title:** Environment selected microbial function rather than taxonomic species in a plateau saline-alkaline wetland

**Authors:** Hongjie Zhang^a,b^, Dayong Zhao^a^, Qinglong L.Wu^b,c,d^, Jin Zeng^b,d,e*^

**Running title:** Microbial community in plateau saline-alkaline wetland

**Institutional affiliations:**

a. State Key Laboratory of Water Disaster Prevention, Hohai University, Nanjing 210098, China

b. State Key Laboratory of Lake and Watershed Science for Water Security, Nanjing Institute of Geography and Limnology, Chinese Academy of Sciences, Nanjing 210008, China

c. Center for Evolution and Conservation Biology, Southern Marine Sciences and Engineering Guangdong Laboratory (Guangzhou), Guangzhou 511458, China

d. Sino-Danish Centre for Education and Research, University of Chinese Academy of Sciences, Beijing 100039, China

e. Poyang Lake Wetland Research Station, Nanjing Institute of Geography and Limnology, Chinese Academy of Sciences, Jiujiang 332899, China

*** Corresponding author:** Jin Zeng, Nanjing Institute of Geography and Limnology, Chinese Academy of Sciences, 73 East Beijing Road, Nanjing 210008, China

E-mail address: jzeng@niglas.ac.cn; Tel.: +86 25 86881090

**SUPPLEMENTARY METHODS**

**Sequence data processing**

Bioinformatics analysis of amplicon sequencing data was performed using QIIME2 (q2, v2021.2.0) (1). The raw sequences were imported and demultiplexed using the PairedEndFastqManifestPhred33 Fastq manifest format, and then the primers were removed from the demultiplexed sequences using Cutadapt (v2021.2.0) (2). Subsequently, the DADA2 pipeline was utilized to denoise the sequences, identify amplicon sequence variants (ASVs), and generate representative sequences and a feature table (3). Taxonomic classification of representative sequences was performed using the q2 feature-classifier classify-sklearn command (4) against the RDPClassifier_16S_trainsetNo18_QiimeFormat database (RDP_classifier_515-806.qza) (5). Feature tables were further filtered to remove the unclassified, Archaea, Chloroplast, Mitochondria and low-frequency (< 0.0005% of the total sequence) ASVs. We used the q2-align-to-tree-mafft-fasttree plugin to align the ASVs (6) and recreate the phylogenies (7). Subsequently, the q2-diversity plugin was utilized to rarefy each feature table, calculate alpha diversity (8), and generate Bray-Curtis distance metrics (9). The relative abundances of feature tables at different taxonomic levels were determined via the q2-feature-table relative-frequency plugin (10).

The raw reads obtained from the metagenomic sequencing were subjected to quality trimming using the Kneaddata pipeline (v0.7.4) to remove adaptors, ambiguous reads, low-quality reads (quality value < 20), and host reads (11, 12). Subsequently, these filtered reads were combined and used for co-assembly into contigs using MEGAHIT (v1.2.9) with "--k-min 29 --k-max 141 --k-step 20" parameters (13). QUAST (v5.0.2) was then used to assess the quality of assembled contigs (14). The open reading frames (ORFs) of the assembled contigs were predicted using Prodigal (v2.6.3) (15). All ORFs were then clustered into a set of unique genes using CD-HIT (parameters: -aS 0.9 -c 0.95 -G 0 -g 0 -T 0 -M 0) (16). The relative abundance (transcripts per million, TPM) of each unique gene were calculated and normalized using Salmon (v1.4.0) (17). The Kraken2 (v2.0.9) with the standard database (NCBI: January 2022) was employed to determine the taxonomic information of each sample (18). To comprehensively investigate the functional genes involved in biogeochemical cycling, the unique genes were subsequently searched against the manually curated methane cycling (MCycDB) (19), nitrogen cycling (NCycDB) (20), and sulfur cycling (SCycDB) (21) database using DIAMOND (v2.1.0.154) with an *e*-value threshold of < 1*e*^-5^ (22, 23).

**Binning and metagenome-assembled genomes (MAGs)**

The metaWRAP pipeline (v1.2.3) was used for genome binning (24), which combines MetaBAT 2 (v2.12.1) (25), MaxBin 2.0 (v2.2.4) (26), and Concoct (v1.0.0) (27) to recover the metagenome-assembled genomes (MAGs) based on tetranucleotide frequencies, coverage, and %GC content. Subsequently, the resulting MAGs were processed into a final bin set using metaWRAP’s bin_refinement module, and the consolidated bin sets were further improved by reassemble_bins module to obtain the MAGs (28). The completeness and contamination of the MAGs were assessed with CheckM (v1.0.12), while low-quality MAGs (completeness < 50% or contamination > 10%) were removed (29). An average nucleotide identity (ANI) of 95% was applied as cutoff to dereplicate MAGs from the secondary ANI comparison using dRep (v2.6.2) (30). The relative abundance of each MAGs was calculated and normalized using CoverM (v0.6.1) (31). GTDB-Tk (v1.5.1) was used to assign the taxonomic information of MAGs and construct the phylogenomic trees (32). The iTOL platform was used to visualize the phylogenomic trees (33). For functional annotation, the ORFs of MAGs were predicted by Prodigal (v2.6.3) and searched against MCycDB, NCycDB, and SCycDB using DIAMOND with an *e*-value threshold of < 1*e*^-5^ (15, 23).

**References**

1. Bolyen E, Rideout JR, Dillon MR, Bokulich N, Abnet CC, Al-Ghalith GA, Alexander H, Alm EJ, Arumugam M, Asnicar F, Bai Y, Bisanz JE, Bittinger K, Brejnrod A, Brislawn CJ, Brown CT, Callahan BJ, Caraballo-Rodriguez AM, Chase J, Cope EK, Da Silva R, Diener C, Dorrestein PC, Douglas GM, Durall DM, Duvallet C, Edwardson CF, Ernst M, Estaki M, Fouquier J, Gauglitz JM, Gibbons SM, Gibson DL, Gonzalez A, Gorlick K, Guo JR, Hillmann B, Holmes S, Holste H, Huttenhower C, Huttley GA, Janssen S, Jarmusch AK, Jiang LJ, Kaehler BD, Bin Kang K, Keefe CR, Keim P, Kelley ST, Knights D, et al. 2019. Reproducible, interactive, scalable and extensible microbiome data science using QIIME 2. Nat Biotechnol 37:852-857. https://doi.org/10.1038/s41587-019-0209-9.

2. Jiao CC, Zhao DY, Zhou TX, Wu QL, Zeng J. 2023. Habitat-specific regulation of bacterial community dynamics during phytoplankton bloom succession in a subtropical eutrophic lake. Water Res 242:120252. https://doi.org/10.1016/j.watres.2023.120252.

3. Callahan BJ, McMurdie PJ, Rosen MJ, Han AW, Johnson AJA, Holmes SP. 2016. DADA2: High-resolution sample inference from Illumina amplicon data. Nat Methods 13:581-583. https://doi.org/10.1038/Nmeth.3869.

4. Bokulich NA, Kaehler BD, Rideout JR, Dillon M, Bolyen E, Knight R, Huttley GA, Caporaso JG. 2018. Optimizing taxonomic classification of marker-gene amplicon sequences with QIIME 2's q2-feature-classifier plugin. Microbiome 6:90. https://doi.org/10.1186/s40168-018-0470-z.

5. Wang Q, Garrity GM, Tiedje JM, Cole JR. 2007. Naive Bayesian classifier for rapid assignment of rRNA sequences into the new bacterial taxonomy. Appl Environ Microb 73:5261-5267. https://doi.org/10.1128/Aem.00062-07.

6. Katoh K, Misawa K, Kuma K, Miyata T. 2002. MAFFT: a novel method for rapid multiple sequence alignment based on fast Fourier transform. Nucleic Acids Res 30:3059-3066. https://doi.org/10.1093/nar/gkf436.

7. Price MN, Dehal PS, Arkin AP. 2009. FastTree: computing large minimum evolution trees with profiles instead of a distance matrix. Mol Biol Evol 26:1641-1650. https://doi.org/10.1093/molbev/msp077.

8. Zhang HJ, Shui J, Li CR, Ma J, He F, Zhao DY. 2024. Diversity, composition, and assembly processes of bacterial communities within per- and polyfluoroalkyl substances (PFAS)-contained urban lake sediments. Sci Total Environ 957: 177625. https://doi.org/10.1016/j.scitotenv.2024.177625.

9. Bray JR, Curtis JT. 1957. An ordination of the upland forest communities of southern wisconsin. Ecol Monogr 27:325-349. https://doi.org/10.2307/1942268.

10. He RJ, Zeng J, Zhao DY, Wang SR, Wu QLL. 2022. Decreased spatial variation and deterministic processes of bacterial community assembly in the rhizosphere of Phragmites australis across the Middle-Lower Yangtze plain. Mol Ecol 31:1180-1195. https://doi.org/10.1111/mec.16298.

11. Bolger AM, Lohse M, Usadel B. 2014. Trimmomatic: a flexible trimmer for Illumina sequence data. Bioinformatics 30:2114-2120. https://doi.org/10.1093/bioinformatics/btu170.

12. Langmead B, Salzberg SL. 2012. Fast gapped-read alignment with Bowtie 2. Nat Methods 9:357-359. https://doi.org/10.1038/Nmeth.1923.

13. Li DH, Liu CM, Luo RB, Sadakane K, Lam TW. 2015. MEGAHIT: an ultra-fast single-node solution for large and complex metagenomics assembly via succinct de Bruijn graph. Bioinformatics 31:1674-1676. https://doi.org/10.1093/bioinformatics/btv033.

14. Gurevich A, Saveliev V, Vyahhi N, Tesler G. 2013. QUAST: quality assessment tool for genome assemblies. Bioinformatics 29:1072-1075. https://doi.org/10.1093/bioinformatics/btt086.

15. Hyatt D, Chen GL, LoCascio PF, Land ML, Larimer FW, Hauser LJ. 2010. Prodigal: prokaryotic gene recognition and translation initiation site identification. BMC Bioinf 11:119. https://doi.org/10.1186/1471-2105-11-119.

16. Fu LM, Niu BF, Zhu ZW, Wu ST, Li WZ. 2012. CD-HIT: accelerated for clustering the next-generation sequencing data. Bioinformatics 28:3150-3152. https://doi.org/10.1093/bioinformatics/bts565.

17. Patro R, Duggal G, Love MI, Irizarry RA, Kingsford C. 2017. Salmon provides fast and bias-aware quantification of transcript expression. Nat Methods 14:417-419. https://doi.org/10.1038/nmeth.4197.

18. Wood DE, Lu J, Langmead B. 2019. Improved metagenomic analysis with Kraken 2. Genome Biol 20:257. https://doi.org/10.1186/s13059-019-1891-0.

19. Qian L, Yu XL, Zhou JY, Gu H, Ding JJ, Peng YS, He Q, Tian Y, Liu JH, Wang SQ, Wang C, Shu LF, Yan QY, He JG, Liu GL, Tu QC, He ZL. 2022. MCycDB: A curated database for comprehensively profiling methane cycling processes of environmental microbiomes. Mol Ecol Resour 22:1803-1823. https://doi.org/10.1111/1755-0998.13589.

20. Tu QC, Lin L, Cheng L, Deng Y, He ZL. 2019. NCycDB: a curated integrative database for fast and accurate metagenomic profiling of nitrogen cycling genes. Bioinformatics 35:1040-1048. https://doi.org/10.1093/bioinformatics/bty741.

21. Yu XL, Zhou JY, Song W, Xu MZ, He Q, Peng YS, Tian Y, Wang C, Shu LF, Wang SQ, Yan QY, Liu JH, Tu QC, He ZL. 2020. SCycDB: A curated functional gene database for metagenomic profiling of sulphur cycling pathways. Mol Ecol Resour 21:924-940. https://doi.org/10.1111/1755-0998.13306.

22. Buchfink B, Xie C, Huson DH. 2015. Fast and sensitive protein alignment using DIAMOND. Nat Methods 12:59-60. https://doi.org/DOI 10.1038/nmeth.3176.

23. Buchfink B, Reuter K, Drost HG. 2021. Sensitive protein alignments at tree-of-life scale using DIAMOND. Nat Methods 18:366-368. https://doi.org/10.1038/s41592-021-01101-x.

24. Uritskiy GV, DiRuggiero J, Taylor J. 2018. MetaWRAP-a flexible pipeline for genome-resolved metagenomic data analysis. Microbiome 6:158. https://doi.org/10.1186/s40168-018-0541-1.

25. Kang DWD, Li F, Kirton E, Thomas A, Egan R, An H, Wang Z. 2019. MetaBAT 2: an adaptive binning algorithm for robust and efficient genome reconstruction from metagenome assemblies. Peerj 7:e7359. https://doi.org/10.7717/peerj.7359.

26. Wu YW, Simmons BA, Singer SW. 2016. MaxBin 2.0: an automated binning algorithm to recover genomes from multiple metagenomic datasets. Bioinformatics 32:605-607. https://doi.org/10.1093/bioinformatics/btv638.

27. Alneberg J, Bjarnason BS, de Bruijn I, Schirmer M, Quick J, Ijaz UZ, Lahti L, Loman NJ, Andersson AF, Quince C. 2014. Binning metagenomic contigs by coverage and composition. Nat Methods 11:1144-1146. https://doi.org/10.1038/Nmeth.3103.

28. Hu RW, Liu SF, Huang WM, Nan Q, Strong PJ, Saleem M, Zhou ZY, Luo ZW, Shu FQ, Yan QY, He ZL, Wang C. 2022. Evidence for assimilatory nitrate reduction as a previously overlooked pathway of reactive nitrogen transformation in estuarine suspended particulate matter. Environ Sci Technol 56:14852-14866. https://doi.org/10.1021/acs.est.2c04390.

29. Parks DH, Imelfort M, Skennerton CT, Hugenholtz P, Tyson GW. 2015. CheckM: assessing the quality of microbial genomes recovered from isolates, single cells, and metagenomes. Genome Res 25:1043-1055. https://doi.org/10.1101/gr.186072.114.

30. Anantharaman K, Brown CT, Hug LA, Sharon I, Castelle CJ, Probst AJ, Thomas BC, Singh A, Wilkins MJ, Karaoz U, Brodie EL, Williams KH, Hubbard SS, Banfield JF. 2016. Thousands of microbial genomes shed light on interconnected biogeochemical processes in an aquifer system. Nat Commun 7:13219. https://doi.org/10.1038/ncomms13219.

31. Jin H, Quan KY, He QW, Kwok LY, Ma T, Li YL, Zhao FY, You LJ, Zhang HP, Sun ZH. 2023. A high-quality genome compendium of the human gut microbiome of Inner Mongolians. Nat Microbiol 8:150-161. https://doi.org/10.1038/s41564-022-01270-1.

32. Chaumeil PA, Mussig AJ, Hugenholtz P, Parks DH. 2020. GTDB-Tk: a toolkit to classify genomes with the genome taxonomy database. Bioinformatics 36:1925-1927. https://doi.org/10.1093/bioinformatics/btz848.

33. Letunic I, Bork P. 2021. Interactive tree of life (iTOL) v5: an online tool for phylogenetic tree display and annotation. Nucleic Acids Res 49:W293-W296. https://doi.org/10.1093/nar/gkab301.

**SUPPLEMENTARY FIGURES**


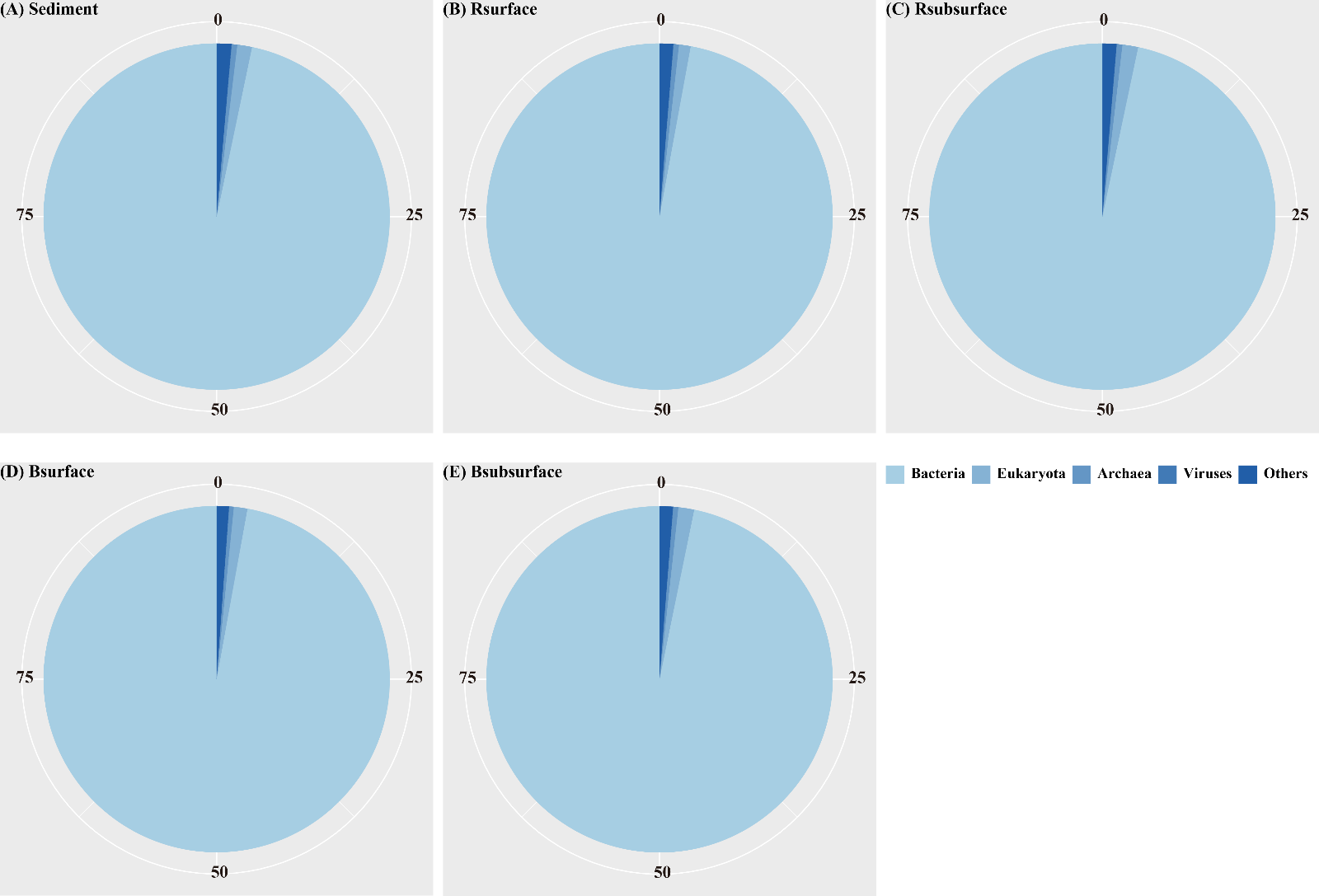


**Fig. S1** Relative abundance of microbial kingdoms across the five habitats. Sediment: the sediment samples; R_surface_, the surface samples of the rhizosphere soils; R_subsurface_, the subsurface samples of the rhizosphere soils; B_surface_, the surface samples of the bulk soils; B_subsurface_, the subsurface samples of the bulk soils.


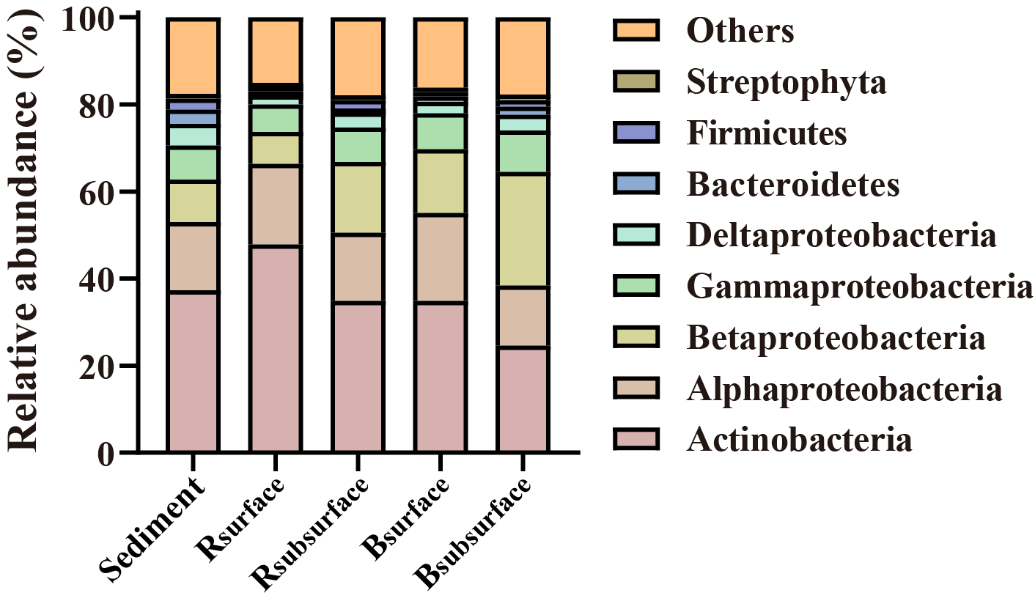


**Fig. S2** Relative abundance of dominant (relative abundance > 1%) phyla/subphyla across the five habitats. Sediment: the sediment samples; R_surface_, the surface samples of the rhizosphere soils; R_subsurface_, the subsurface samples of the rhizosphere soils; B_surface_, the surface samples of the bulk soils; B_subsurface_, the subsurface samples of the bulk soils.


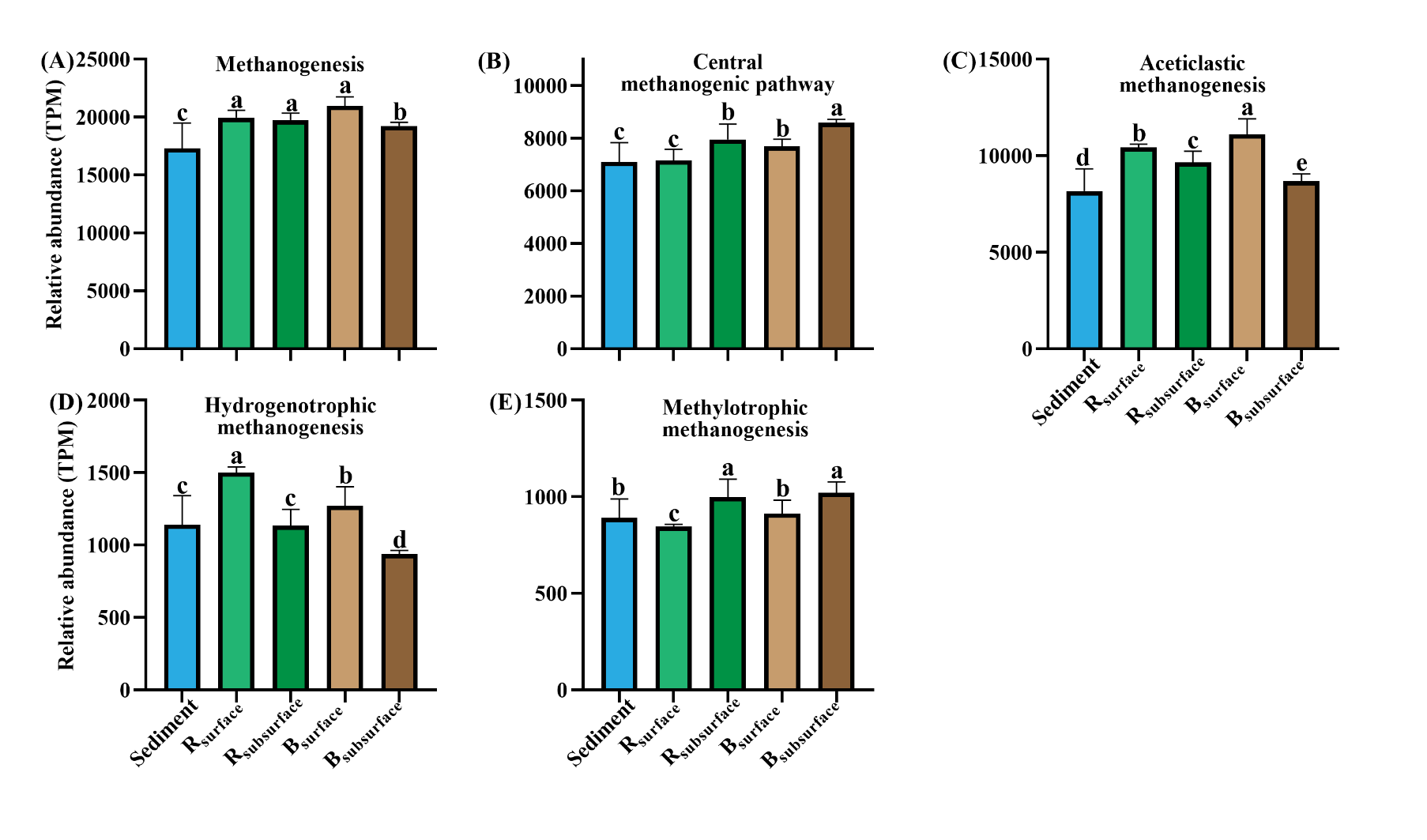


**Fig. S3** Relative abundance of the key pathways involved in methanogenesis. Different letters above the boxes indicated significant difference (*P* < 0.05) among habitats according to the one-way ANOVA. Sediment: the sediment samples; R_surface_, the surface samples of the rhizosphere soils; R_subsurface_, the subsurface samples of the rhizosphere soils; B_surface_, the surface samples of the bulk soils; B_subsurface_, the subsurface samples of the bulk soils.


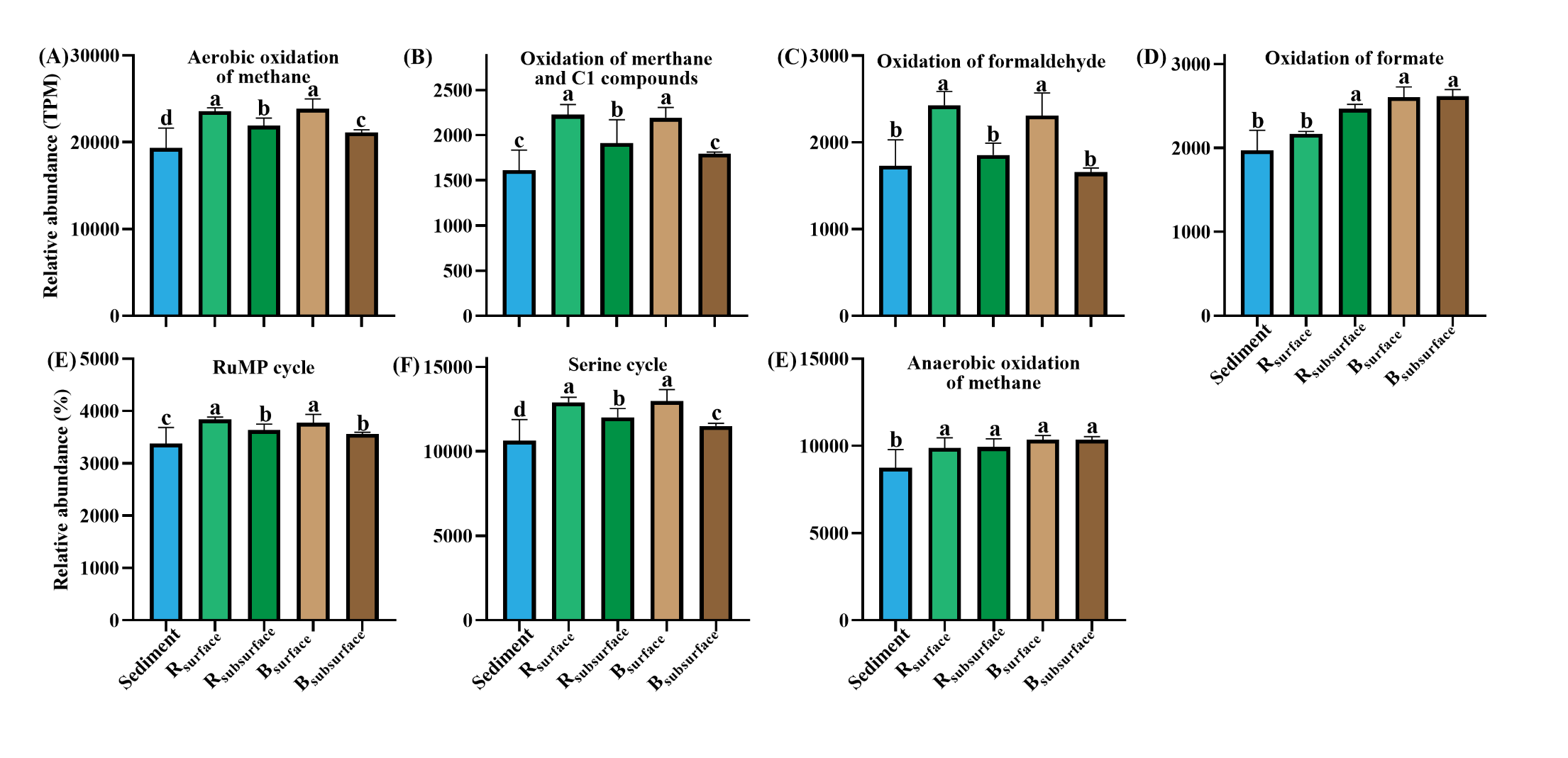


**Fig. S4** Relative abundance of the key pathways involved in oxidation of methane. Different letters above the boxes indicated significant difference (*P* < 0.05) among habitats according to the one-way ANOVA. Sediment: the sediment samples; R_surface_, the surface samples of the rhizosphere soils; R_subsurface_, the subsurface samples of the rhizosphere soils; B_surface_, the surface samples of the bulk soils; B_subsurface_, the subsurface samples of the bulk soils. RuMP cycle, ribulose monophosphate cycle.


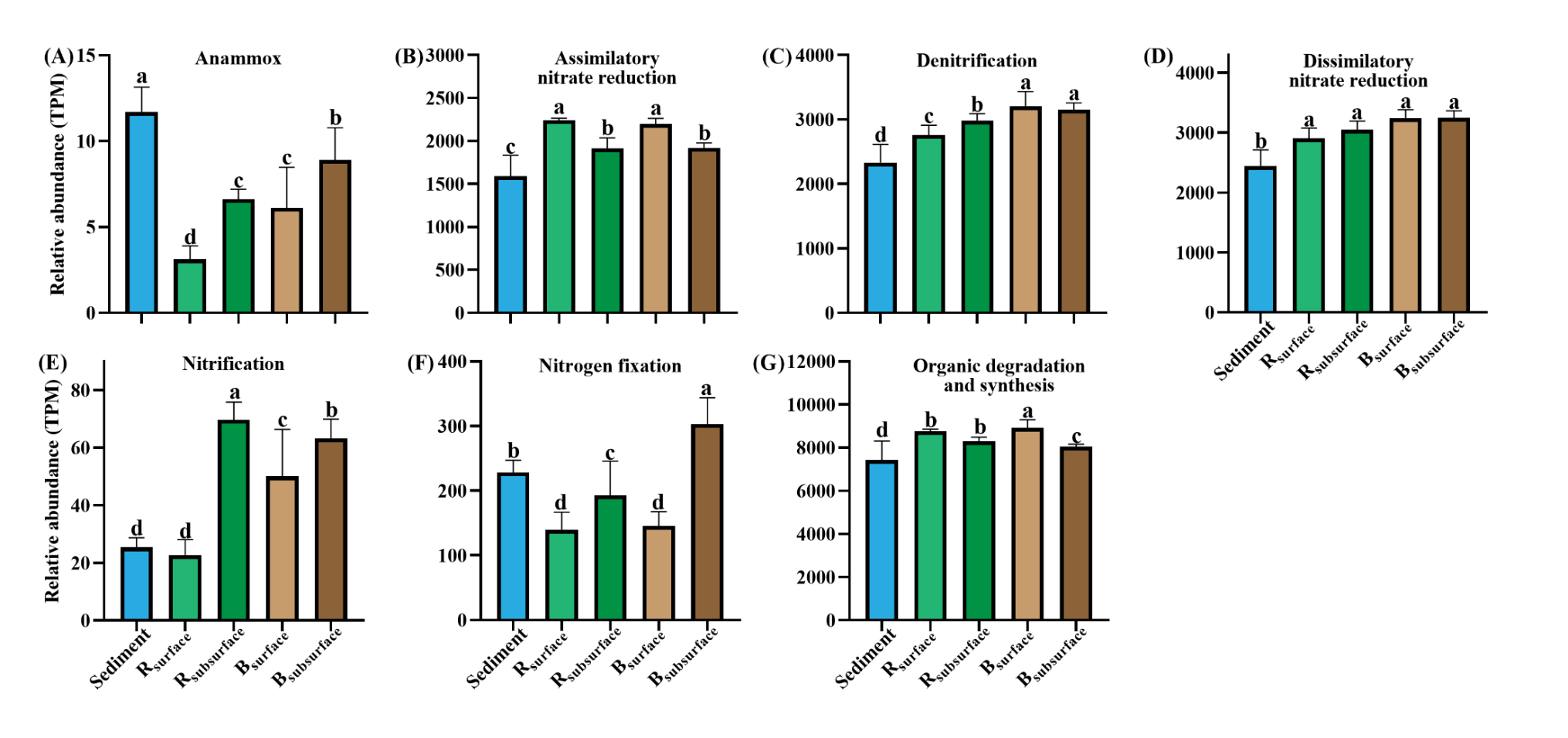


**Fig. S5** Relative abundance of the key pathways involved in nitrogen cycle. Different letters above the boxes indicated significant difference (*P* < 0.05) among habitats according to the one-way ANOVA. Sediment: the sediment samples; R_surface_, the surface samples of the rhizosphere soils; R_subsurface_, the subsurface samples of the rhizosphere soils; B_surface_, the surface samples of the bulk soils; B_subsurface_, the subsurface samples of the bulk soils.


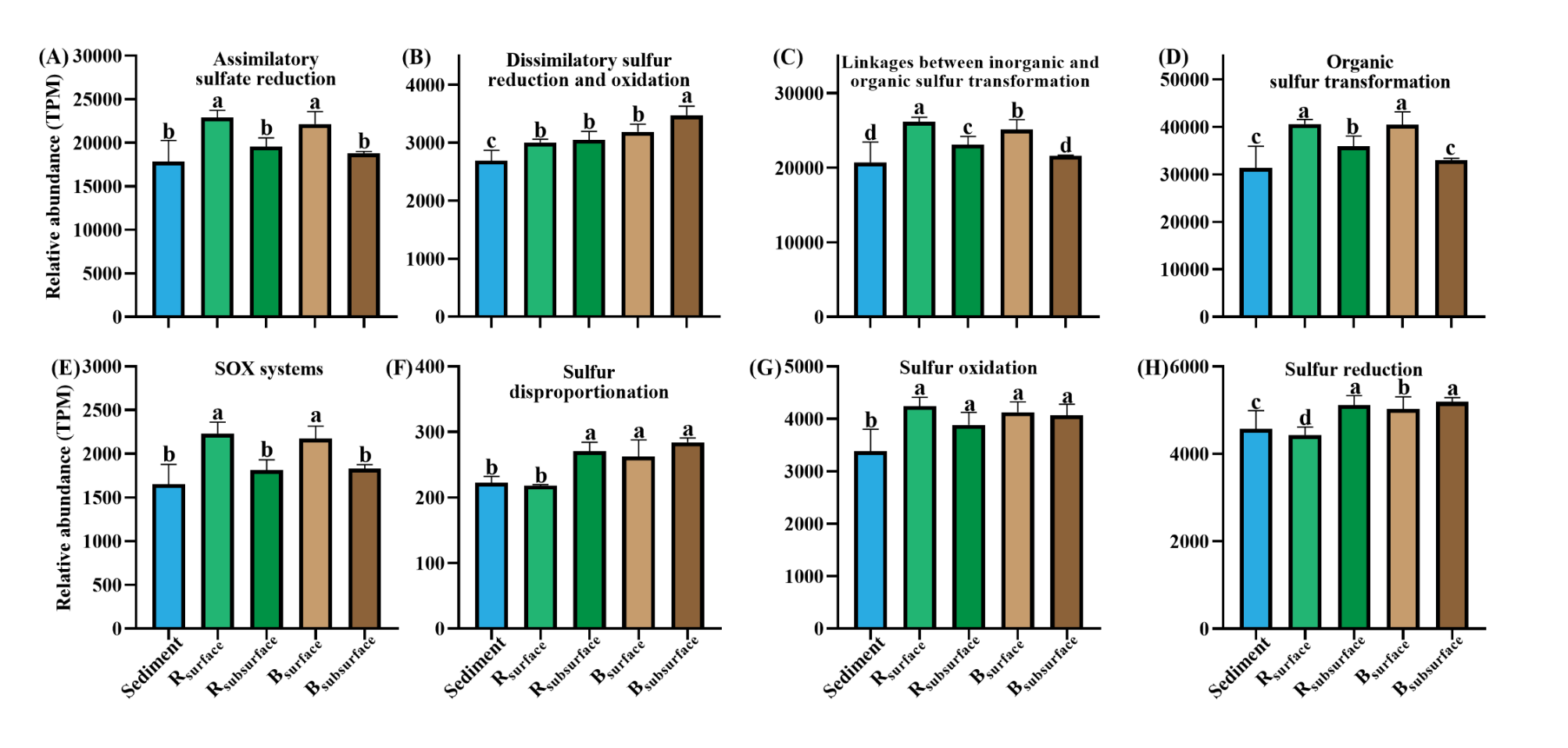


**Fig. S6** Relative abundance of the key pathways involved in sulfur cycle. Different letters above the boxes indicated significant difference (*P* < 0.05) among habitats according to the one-way ANOVA. Sediment: the sediment samples; R_surface_, the surface samples of the rhizosphere soils; R_subsurface_, the subsurface samples of the rhizosphere soils; B_surface_, the surface samples of the bulk soils; B_subsurface_, the subsurface samples of the bulk soils.


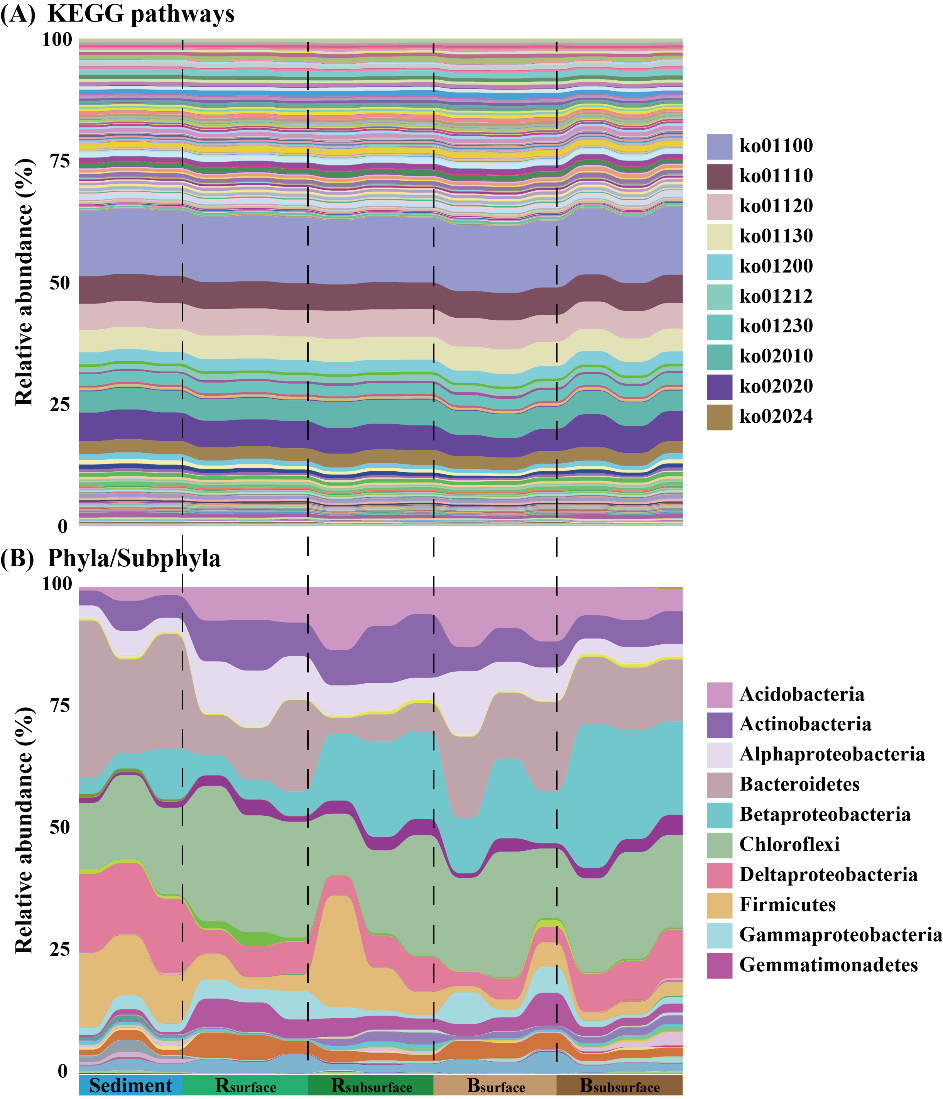


**Fig. S7** The relative abundance of KEGG pathways (A) and phyla/subphyla (B) in different habitats and layers, derived from 16S rRNA sequencing data. The legend displays only the top ten pathways/phyla with the highest relative abundance, detailed information can be seen in Table S5~Table S6 (Supplementary material2). Sediment: the sediment samples; R_surface_, the surface samples of the rhizosphere soils; R_subsurface_, the subsurface samples of the rhizosphere soils; B_surface_, the surface samples of the bulk soils; B_subsurface_, the subsurface samples of the bulk soils.


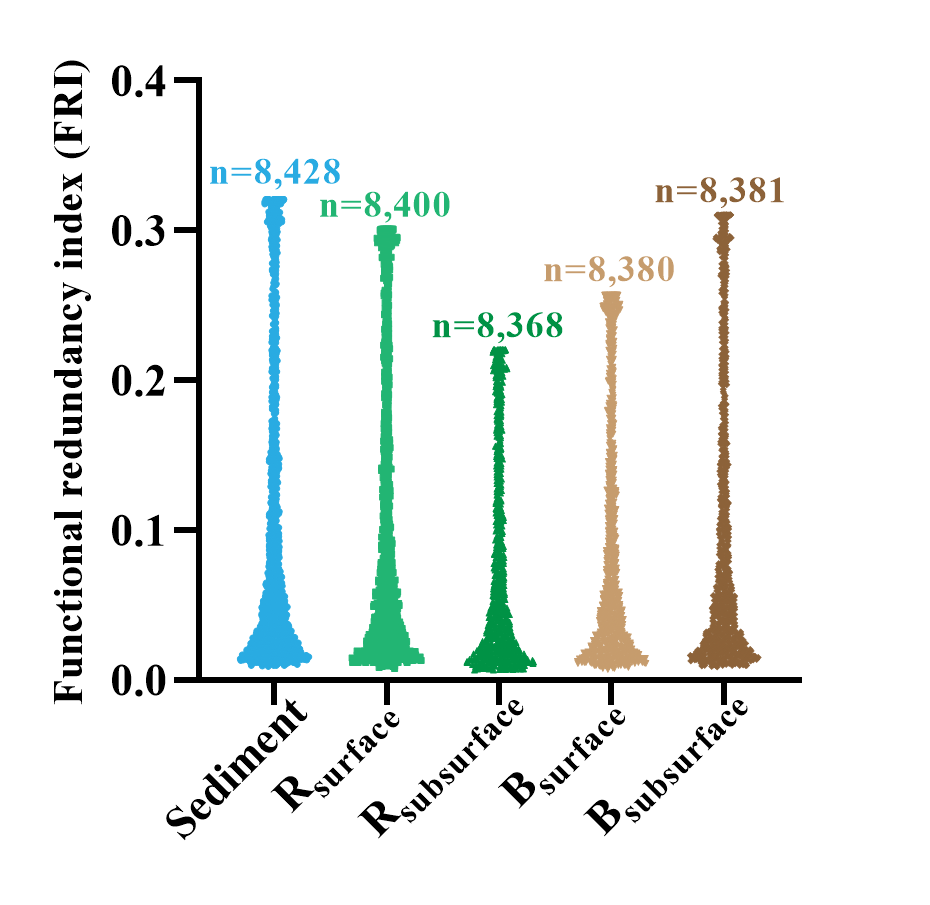


**Fig. S8** Functional redundancy index (FRI) of each KEGG Orthology (KO) across the five habitats. Only KOs with FRI > 0.01 were shown; detailed information can be seen in Table S7 (Supplementary material2). Sediment: the sediment samples; R_surface_, the surface samples of the rhizosphere soils; R_subsurface_, the subsurface samples of the rhizosphere soils; B_surface_, the surface samples of the bulk soils; B_subsurface_, the subsurface samples of the bulk soils.


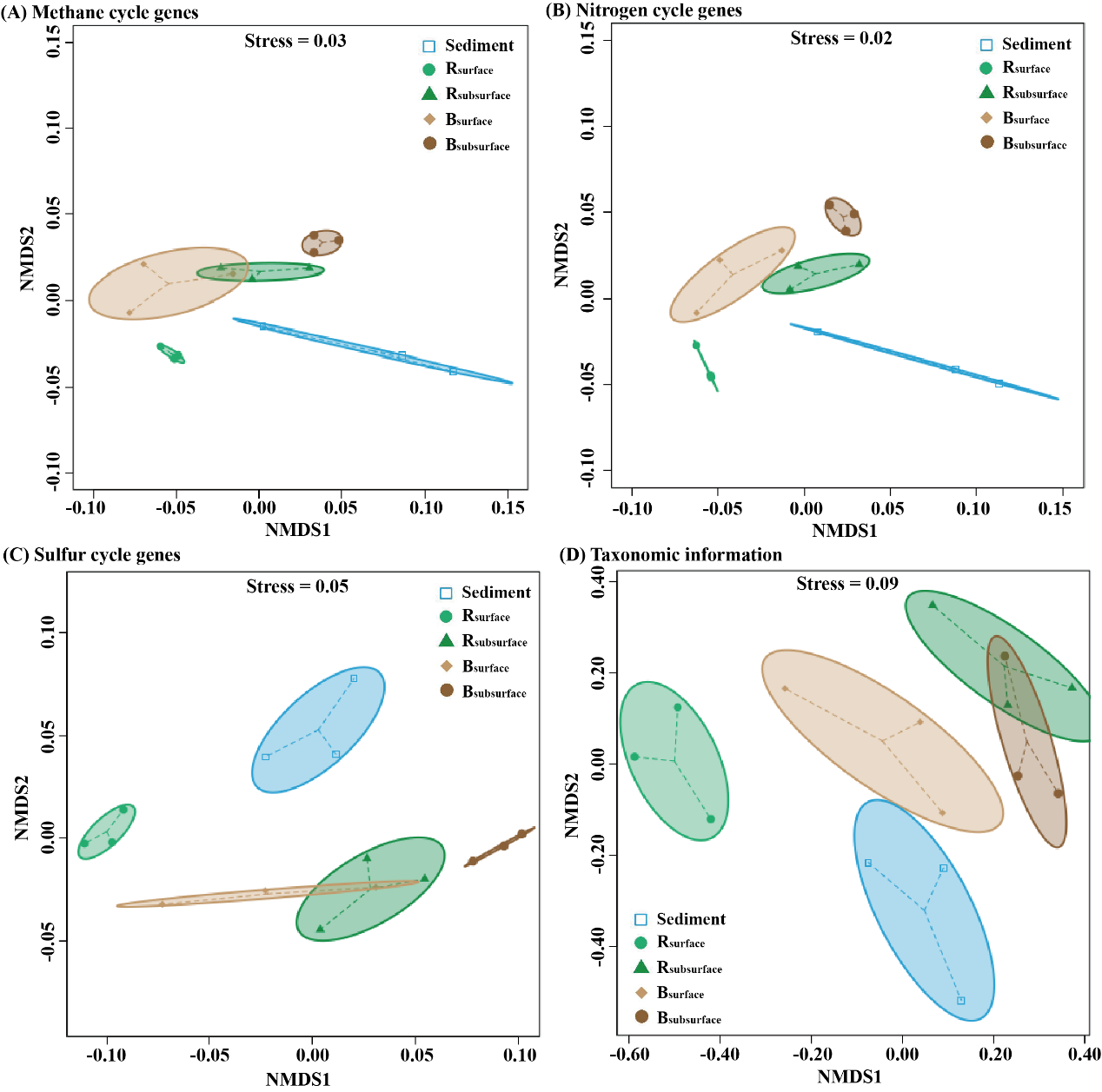


**Fig. S9** Nonmetric multidimensional scaling (NMDS) plots of the functional gene-based (A, methane cycle genes; B, nitrogen cycle genes; C, sulfur cycle genes) and taxonomy-based (D) Bray-Curtis dissimilarities of microbial community composition among the five habitats, derived from metagenomic sequencing data. Sediment: the sediment samples; R_surface_, the surface samples of the rhizosphere soils; R_subsurface_, the subsurface samples of the rhizosphere soils; B_surface_, the surface samples of the bulk soils; B_subsurface_, the subsurface samples of the bulk soils.


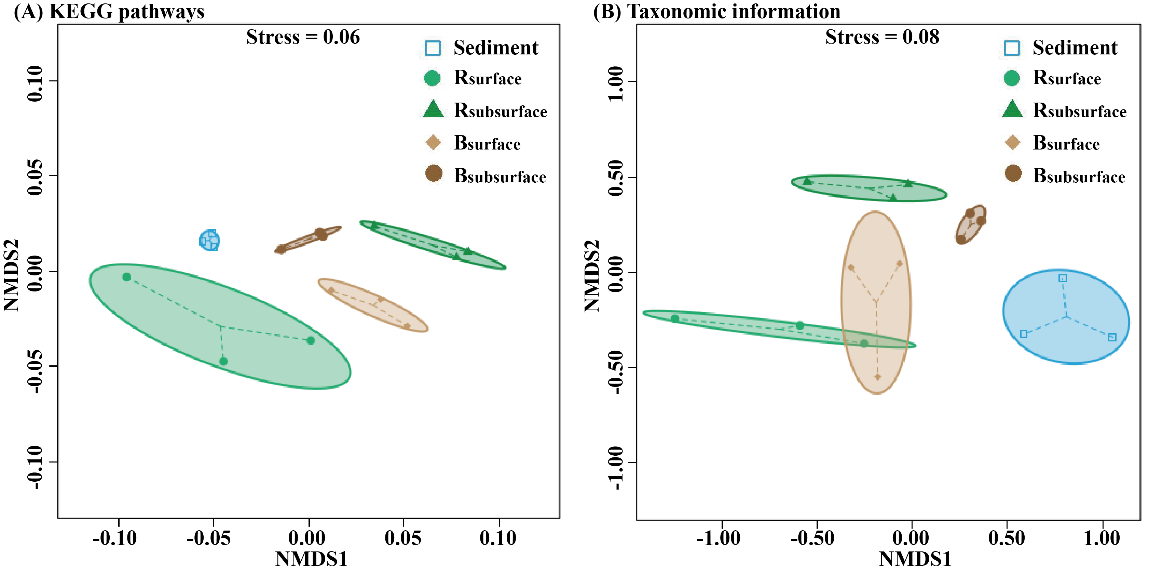


**Fig. S10** Nonmetric multidimensional scaling (NMDS) plots of the Bray-Curtis dissimilarities based on KEGG pathways (A) and taxonomic compositions (B) of microbial community among the five habitats, derived from 16S rRNA sequencing data. Sediment: the sediment samples; R_surface_, the surface samples of the rhizosphere soils; R_subsurface_, the subsurface samples of the rhizosphere soils; B_surface_, the surface samples of the bulk soils; B_subsurface_, the subsurface samples of the bulk soils.


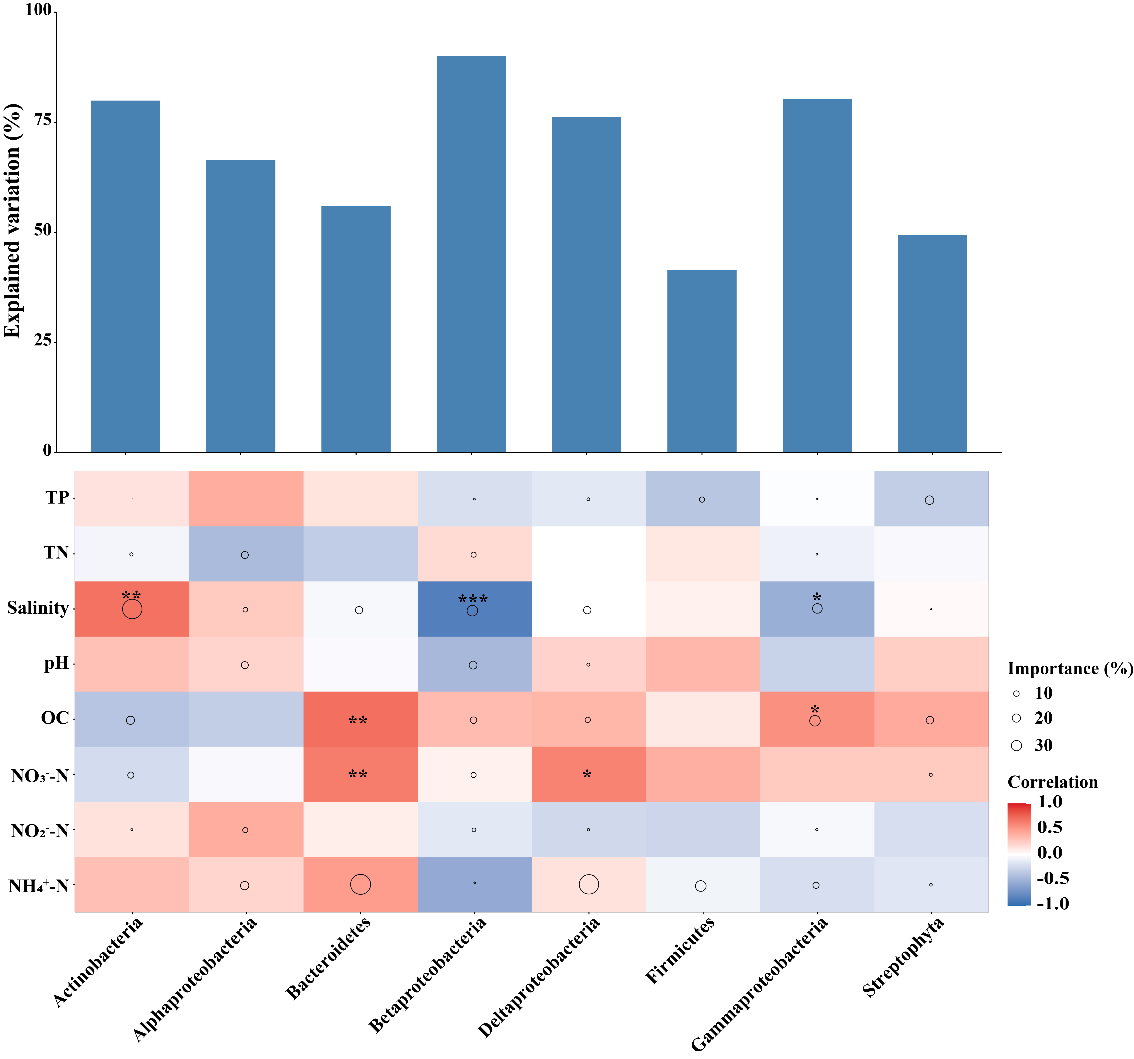


**Fig. S11** Variations in dominant phyla (relative abundance > 1%) explained by different environmental factors, based on correlation and the best multiple regression model. Circle size represents the variable importance (that is, proportion of explained variability calculated via multiple regression modeling and variance decomposition analysis). Colors and asterisk represent Spearman correlations; *: *P* < 0.05; **: *P* < 0.01. TP, total phosphorus; TN, total nitrogen; OC, organic carbon; NO_3_^-^-N, nitrate nitrogen; NO_2_^-^-N, nitrite nitrogen; NH_4_^+^-N, ammonia nitrogen.





**Fig. S12** The relative importance of deterministic and stochastic processes in shaping taxonomic and functional compositions (methane, nitrogen, and sulfur) of microbial community inferred from metagenomic sequencing data using the Null model analysis. Tax, the results of the Null model based on the taxonomic composition of microbial community; CH_4_, the results of the Null model based on the functional composition of methane cycling; N, the results of the Null model based on the functional composition of nitrogen cycling; S, the results of the Null model based on the functional composition of sulfur cycling. Sediment: the sediment samples; R_surface_, the surface samples of the rhizosphere soils; R_subsurface_, the subsurface samples of the rhizosphere soils; B_surface_, the surface samples of the bulk soils; B_subsurface_, the subsurface samples of the bulk soils.





**Fig. S13** The relative importance of deterministic and stochastic processes in shaping taxonomic and functional compositions (KEGG pathways) of microbial community inferred from 16S rRNA sequencing data using the Null model analysis. Tax, the results of the Null model based on the taxonomic composition of microbial community; ko, the results of the Null model based on the KEGG pathways. Sediment: the sediment samples; R_surface_, the surface samples of the rhizosphere soils; R_subsurface_, the subsurface samples of the rhizosphere soils; B_surface_, the surface samples of the bulk soils; B_subsurface_, the subsurface samples of the bulk soils.


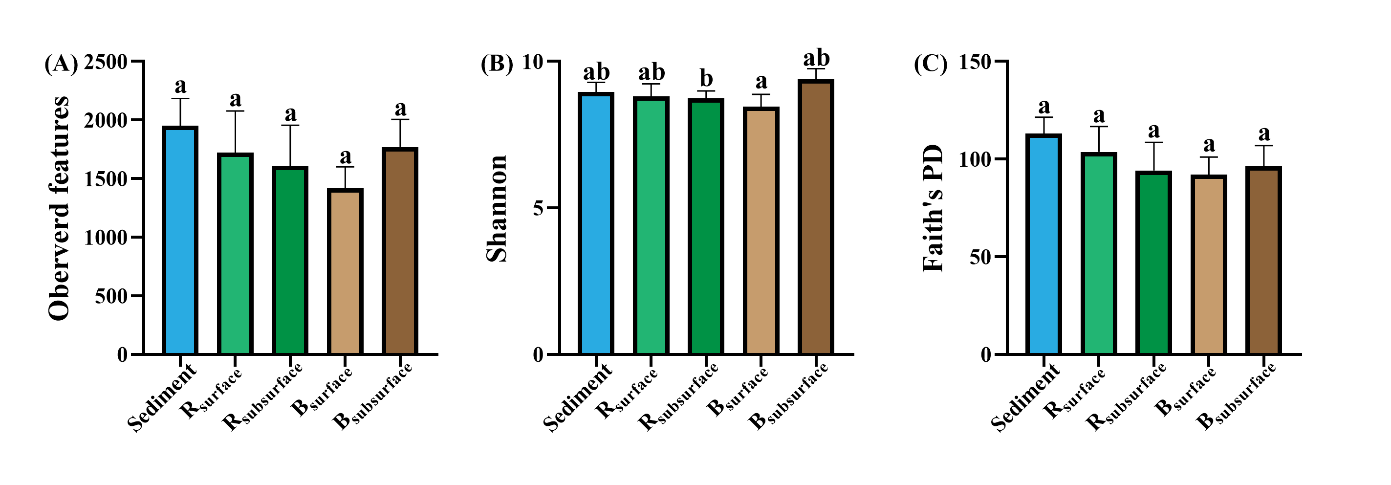


**Fig. S14** Alpha diversity of microbial communities, including observed features (A), Shannon index (B), and Faith's PD (C), across sediment, rhizosphere soils, and bulk soils based on 16S rRNA gene data. Different letters above the boxes indicated significant difference (*P* < 0.05) among habitats according to the one-way ANOVA. Sediment: the sediment samples; R_surface_, the surface samples of the rhizosphere soils; R_subsurface_, the subsurface samples of the rhizosphere soils; B_surface_, the surface samples of the bulk soils; B_subsurface_, the subsurface samples of the bulk soils.


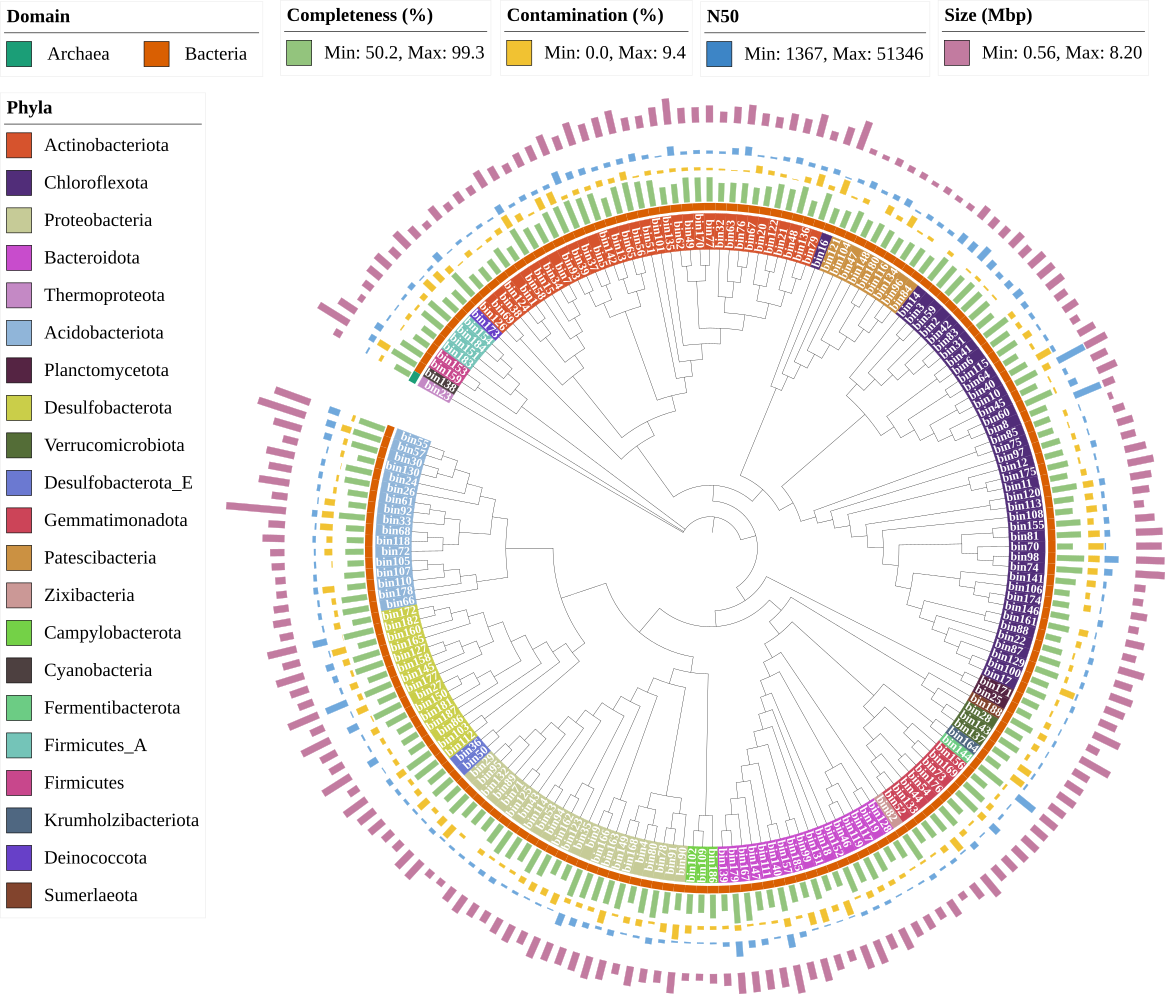


**Fig. S15** Phylogenomic tree for metagenomic assembled genomes (MAGs) based on the concatenated alignments of marker genes from GTDB-Tk.


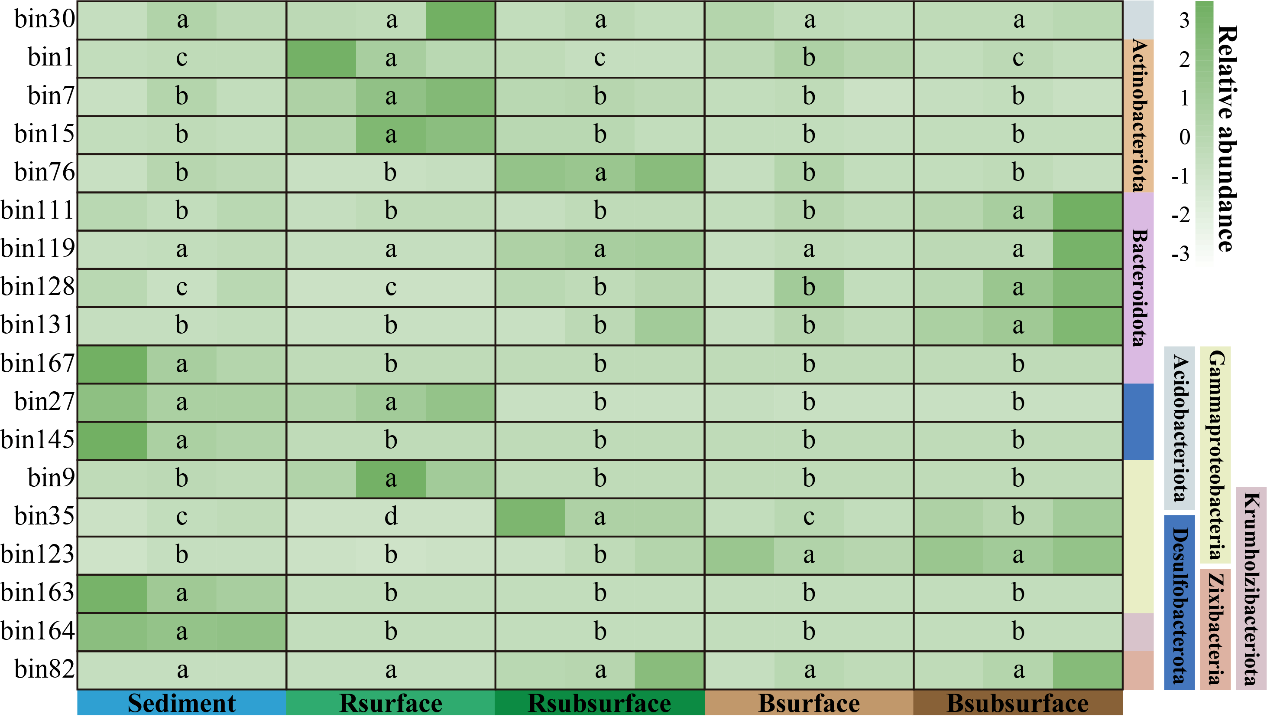


**Fig. S16** Heatmaps of normalized relative abundances of the 18 metagenomic assembled genomes (MAGs), which contained the *nod* gene. The normalization process involved subtracting the mean of each row from each value and then dividing it by the standard deviation of that row. Sediment: the sediment samples; R_surface_, the surface samples of the rhizosphere soils; R_subsurface_, the subsurface samples of the rhizosphere soils; B_surface_, the surface samples of the bulk soils; B_subsurface_, the subsurface samples of the bulk soils. Different letters indicated significant difference (*P* < 0.05) among habitats according to the one-way ANOVA.
